# Rice transcription factor OsMADS25 modulates root growth and confers salinity tolerance via the ABA–mediated regulatory pathway and ROS scavenging

**DOI:** 10.1371/journal.pgen.1007662

**Published:** 2018-10-10

**Authors:** Ning Xu, Yanli Chu, Hongli Chen, Xingxing Li, Qi Wu, Liang Jin, Guixue Wang, Junli Huang

**Affiliations:** Key Laboratory of Biorheological Science and Technology, Ministry of Education, Bioengineering College, Chongqing University, Chongqing, P. R. China; Wake Forest University, UNITED STATES

## Abstract

Plant roots are constantly exposed to a variety of abiotic stresses, and high salinity is one of the major limiting conditions that impose constraints on plant growth. In this study, we describe that *OsMADS25* is required for the root growth as well as salinity tolerance, via maintaining ROS homeostasis in rice (*Oryza sativa*). Overexpression of *OsMADS25* remarkably enhanced the primary root (PR) length and lateral root (LR) density, whereas RNAi silence of this gene reduced PR elongation significantly, with altered ROS accumulation in the root tip. Transcriptional activation assays indicated that OsMADS25 activates *OsGST4* (glutathione *S*–transferase) expression directly by binding to its promoter. Meanwhile, *osgst4* mutant exhibited repressed growth and high sensitivity to salinity and oxidative stress, and recombinant OsGST4 protein was found to have ROS–scavenging activity *in vitro*. Expectedly, overexpression of *OsMADS25* significantly enhanced the tolerance to salinity and oxidative stress in rice plants, with the elevated activity of antioxidant enzymes, increased accumulation of osmoprotective solute proline and reduced frequency of open stoma. Furthermore, OsMADS25 specifically activated the transcription of *OsP5CR*, a key component of proline biosynthesis, by binding to its promoter. Interestingly, overexpression of *OsMADS25* raised the root sensitivity to exogenous ABA, and the expression of ABA–dependent stress–responsive genes was elevated greatly in overexpression plants under salinity stress. In addition, OsMADS25 seemed to promote auxin signaling by activating *OsYUC4* transcription. Taken together, our findings reveal that *OsMADS25* might be an important transcriptional regulator that regulates the root growth and confers salinity tolerance in rice via the ABA–mediated regulatory pathway and ROS scavenging.

## Author summary

Plant roots are constantly exposed to a variety of abiotic stresses, and high salinity is one of major limiting conditions that impose constraints on plant growth. Here, we show that transcription factor OsMADS25 positively regulates the root system development and tolerance to salinity and oxidative stress in rice plants. We also provide strong evidence that OsMADS25 increases the ROS-scavenging capacity and proline accumulation by activating the expression of *OsGST4* and *OsP5CR* directly. Moreover, *OsMADS25* promotes ABA–dependent abiotic stress–responsive regulatory pathway. In addition, OsMADS25 seems to promote auxin signaling by activating *OsYUC4* transcription. Overall, enhanced antioxidant responses and proline accumulation via the ABA–mediated regulatory pathway, have been proposed to be crucial for OsMADS25 to regulate the salinity tolerance in rice plants.

## Introduction

Rapid root growth is due largely to rapid elongation of cells in plant root. After cell division, newly formed cells begin the process of elongating and differentiation [1], which is regulated by reactive oxygen species (ROS) homeostasis [2]. ROS are important signaling molecules that affect many aspects of plant development such as the cell cycle, programmed cell death, hormone signaling as well as the response to environmental stresses [3–6], and disruption in ROS signaling leads to defects in these developmental processes [7].

Plant roots are constantly exposed to a variety of abiotic stresses, and high salinity is one of major limiting conditions that impose constraints on plant root growth, as a stress that encourages ROS production. In plants, apoplastic ROS, mainly generated by NADPH oxidase (Respiratory Burst Oxidase Homologs, Rbohs), are toxic to plant cells at high concentrations and ROS homeostasis must be strictly controlled by a delicate balance between ROS–producing and–scavenging enzymes [8]. To minimize or prevent oxidative damage to cells by ROS and to maintain cellular redox homeostasis, plants have evolved defense systems that include ROS–scavenging enzymes, such as ascorbate peroxidase (APX), superoxide dismutase (SOD), catalase (CAT) and glutathione peroxidase (GPX) [9]. Various studies have shown that the ROS–scavenging enzymes are involved in tolerance to abiotic stress in plants [4,10,11]. A gain–of–function mutant of *Arabidopsis* in which *APX2* is constitutively overexpressed shows drought tolerance and exhibits an improved efficiency of water use [12]. Transgenic *Arabidopsis* plants overexpressing *OsAPXa* or *OsAPXb* exhibit increased tolerance to salinity stress [13]. Constitutive expression of *OsGSTU4* (glutathione *S*–transferase) in *Arabidopsis* improves the tolerance to salinity and oxidative stress [14]. Although the balance between ROS production and detoxification is shown to be modulated by a large and complicated network, the precise mechanism underlying ROS homeostasis in response to abiotic stresses in plants remains unclear.

Proline has been considered as a multi–functional molecule in plants, and numerous studies have demonstrated that the proline accumulation is enhanced in response to different abiotic stresses [15–17]. In plants, proline is synthesized by the pyrroline–5–carboxylate synthetase (*P5CS*) and P5C reductase (*P5CR*) [18], which are induced by osmotic and salt stresses and also activated by ABA [17,19]. As an osmoprotective molecule, proline is recognized to protect cells against osmotic stress by increasing their antioxidant enzyme capacity, suggesting its ROS–scavenging activity and acting as a singlet oxygen quencher [20]. Exogenous proline treatment reduced Hg^2+^ toxicity in rice through scavenging ROS [21]. Correspondingly, ROS accumulation was reduced in overexpression–*P5CS* transgenic tobacco plants, in which proline levels were enhanced by accelerating of the proline biosynthetic pathway [22,23]. By contrast, reduced proline levels in *p5cs1* insertion mutants caused accumulation of ROS and elevated oxidative damage [24].

Abscisic acid (ABA) is a key regulator of both plant development and stress responses, and has been shown to regulate a range of physiological processes including seed germination and dormancy, root development, stomatal movements and tolerance to abiotic stresses [25–28]. ABA dramatically accumulates under osmotic stress such as drought and salinity, and plays pivotal roles in the stress responses by modulating the gene expression and cellular processes [26,29]. There is convincing evidence that ROS are also integral parts of ABA signaling networks [30,31]. In parallel to pattern–triggered ROS production, ABA also regulates ROS generation through plasma membrane–localized NADPH oxidases [32]. These ROS, as important second messengers, are involved in ABA signaling pathway to regulate the physiological processes [29,30]. In *Arabidopsis*, a double loss–of–function mutant *rbohD*/*rbohF* impairs ABA–induced H_2_O_2_ production and stomatal closing, and ABA–mediated inhibition of root growth [32]. In drought stress, ABA induces stomatal closure by stimulating ROS production in guard cells by guard cell–specific NADPH oxidases [26]. 2C–type protein phosphatase ABA INSENSITIVE2 (ABI2) interacts with GPX3 to regulate the redox state of guard cells in ABA and drought stress responses [33]. ABI1 binds to phosphatidic acid which is produced by phospholipase Da1, and phosphatidic acid interacts with RBOHD/F, stimulating ROS production [34]. Mutation of *Phospholipase Da1* (*PLDa1*) failed to produce ROS in guard cells in response to ABA [35]. Thus, ROS are important second messengers in ABA signaling in guard cells.

MADS–box transcription factors have been shown play crucial roles in the regulation of developmental processes [36,37]. Recently, there are convincing reports that MADS–box genes also participate in the regulation of hormones and the abiotic stress response. In *Arabidopsis*, the mutation of flowering–time gene *SOC1* confers freezing tolerance [38], and *AGL21* modulates osmotic stress tolerance by controlling the expression of *ABI5* [39]. In rice, *OsMADS26* negatively regulates the resistance to pathogens and drought tolerance [40], while *OsMADS87* is involved in heat sensitivity [41]. Despite the recent progress that *OsMADS25* regulates the root system development in rice in a nitrate–dependent manner [42], its function in abiotic stress remains elusive. In this current work, we aim to reveal the regulatory roles and possible mechanism of *OsMADS25* in response to salinity stress in rice.

## Results

### Overexpression of *OsMADS25* reduces the ROS levels in the roots in rice

**Fig 1:**
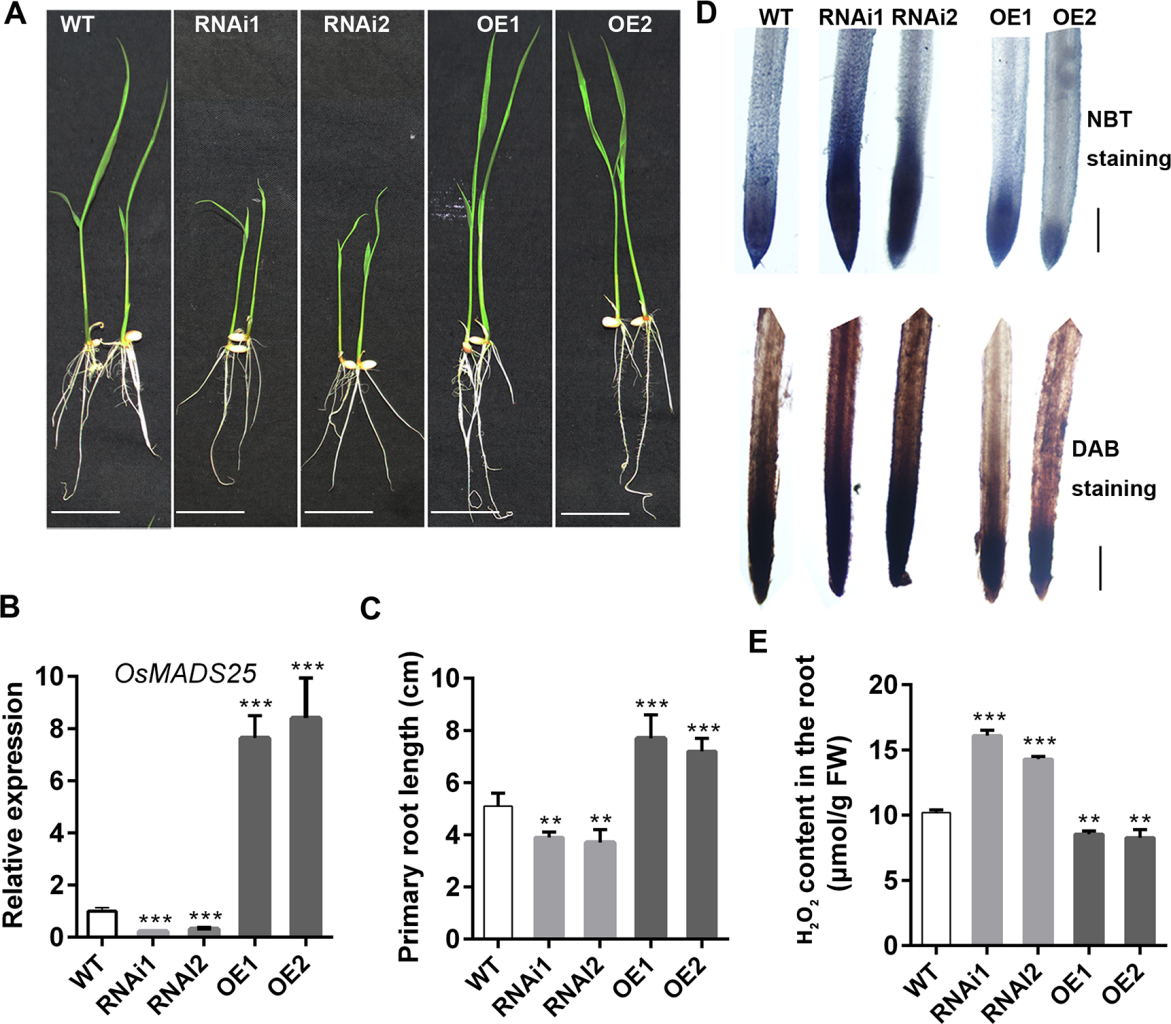
Root growth of *OsMADS25* transgenic lines is correlated with the ROS levels. **A.** Seedlings of wild type and *OsMADS25* transgenic lines grown in standard 1/2 MS medium for 5 days. Scale bars, 2 cm. **B.** Relative transcript levels of *OsMADS25* in *OsMADS25* transgenic lines by qPCR analysis. **C.** Primary root length of 5–day–old wild type and *OsMADS25* transgenic seedlings. **D.** NBT and DAB staining for O_2_^–^ and H_2_O_2_, respectively, in the root tips of wild type and *OsMADS25* transgenic lines shown in image A. Scale bars, 1 mm. **E.** Quantification of H_2_O_2_ content in the roots of wild type and *OsMADS25* transgenic plants shown in image A. WT, wild type. RNAi1 and RNAi2, *OsMADS25*–RNAi transgenic lines. OE1 and OE2, *OsMADS25* overexpression transgenic lines. NBT, nitroblue tetrazolium. DAB, 3, 3’–diaminobenzidine. Three independent experiments were performed with similar results. Data are means ± SE (n = 10). The statistical significance of the measurements using one-way analysis of variance (ANOVA) was determined using Student’s *t*-test. Asterisks indicate the significant difference between *OsMADS25* transgenic lines and WT plants (*t*–test, ^*^P < 0.05, ^**^P < 0.01 or ^***^P < 0.001).

*OsMADS25* is preferentially expressed in the root during the growth period (S1A Fig). Here, independent *OsMADS25*–overexpression transgenic lines (OE1 and OE2) and–RNAi lines (RNAi1 and RNAi2) with significantly up–or down–regulated mRNA levels of *OsMADS25* were produced, and the root system of *OsMADS25* transgenic lines was indicated to change greatly in standard 1/2 MS medium, compared with their wild–type counterparts (Fig 1A and 1B). Overexpression of *OsMADS25* remarkably enhanced the primary root (PR) length, whereas RNAi silence of this gene reduced PR elongation (Fig 1C). The root elongation is inhibited by ROS via enhancing *Rbohs* expression, and silencing of *RbohC* accelerates root elongation [7]. To test whether the roots of *OsMADS25* transgenic lines have altered ROS levels, we used the ROS–reactive dyes NBT and DAB to detect the levels of O_2_^–^ and H_2_O_2_ in PRs, respectively. We found that OsMADS25–OE roots had the strong staining just in the first 1 mm of the root tip of PR, and the staining intensity started to decrease in the differentiation zone and elongation zone behind the root meristem, with only weak staining observed at a distance of 3 mm from the root tip (Fig 1D). OsMADS25–RNAi roots also showed strong staining in the root tip as in the wild type; but the staining continued to be strong, and was also detected in the differentiation zone and elongation zone of the root tip (Fig 1D), due to an increase of H_2_O_2_ content in roots, compared to wild type (Fig 1E).

In addition, *OsMADS25* also greatly affected lateral root (LR) density (S1B Fig). The LR formation is proposed to be divided into the initiation and emergence phases primarily [43]. Further investigations on the developmental basis of LR by methylene blue staining showed that the density of lateral root primordia (LRPs) was significantly increased in OsMADS25–OE plants, whereas remarkably reduced in OsMADS25–RNAi lines, compared to that in wild type (S1C and S1D Fig), which suggests that *OsMADS25* might control LRP initiation in rice. We also detected ROS accumulation in LRPs by DAB and NBT staining, respectively, and there seemed to be no difference between *OsMADS25* transgenic lines and wild type (S2 Fig). Indeed, *OsMADS25* also significantly affected shoot growth (S1E Fig). As expected, less ROS accumulation was detected in 7–day–old shoots, mature leaves, as well as bracts of OsMADS2*5*–OE lines, but increased levels of ROS were deposited in the counterparts of OsMADS2*5*–RNAi plants, compared to wild type (S1F–S1H Fig). Together, these data indicate that *OsMADS25* is required for maintaining cellular redox homeostasis, and down–regulated expression of *OsMADS25* enhances ROS accumulation, especially in the differentiation zone and elongation zone of the root tip, which might affect the root elongation.

### Overexpression of *OsMADS25* enhances root cell elongation

**Fig 2:**
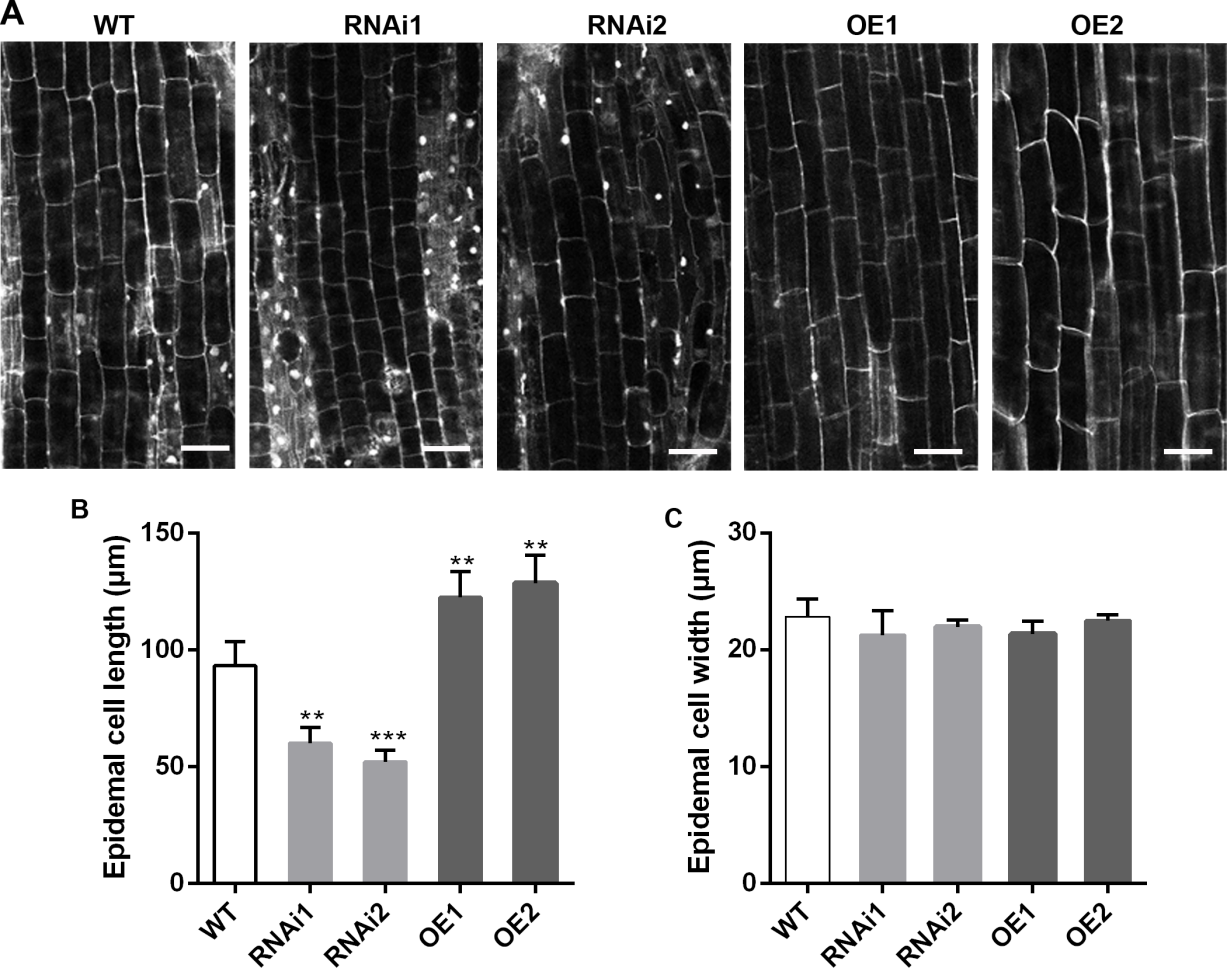
Overexpression of *OsMADS25* enhances the cell elongation. **A.** Propidium iodide (PI)–stained root epidermal cells of 5–day–old wild type and *OsMADS25* transgenic roots grown in standard 1/2 MS medium. Scale bars, 50 μm. **B** and **C.** Average epidermal cell length and cell width from 5–day–old wild type and *OsMADS25* transgenic roots shown in image A, respectively. To measure cell length, germinated seeds were grown in standard 1/2 MS medium for 5 days, and the roots were stained with PI followed by washing for twice with sterile water, then imaged with a Leica SP8 confocal microscope. Epidermal cell length was averaged from at least 50 cells per root at a distance of 1 cm from the root tips from at least five roots examined for each treatment. Cell length was measured using Leica SP8 software. WT, wild type. RNAi1 and RNAi2, *OsMADS25*–RNAi transgenic lines. OE1 and OE2, *OsMADS25* overexpression transgenic lines. Three independent experiments were performed. The statistical significance of the measurements using one-way analysis of variance (ANOVA) was determined using Student’s *t*–test. Asterisks indicate the significant difference between *OsMADS25* transgenic lines and WT plants (^*^P < 0.05, ^**^P < 0.01 or ^***^P < 0.001).

ROS are important signaling molecules that regulate the root growth and development [6,44]. We wonder whether the root system architecture of *OsMADS25* transgenic lines in standard 1/2 MS medium is the result of alteration in cell elongation. Then we investigated the epidermal cell length at day 5, when there was significant difference in the root elongation between transgenic lines and wild type in standard 1/2 MS medium (Fig 2A). To ensure that we were measuring comparable cells in wild type and *OsMADS25* transgenic lines, the cells we measured were at the same distance, 8–10 mm from the root tip in the mature zone. We found that, at this developmental stage, OsMADS25–RNAi roots had significantly shorter cells, but OsMADS25–OE roots had remarkably longer cells than wild type (Fig 2B). At the same distance from the root tip, the epidermal cell length was reduced more than 30% in OsMADS25–RNAi roots, whereas increased over 40% in OsMADS25–OE lines, compared to their wild–type counterparts (Fig 2B). No significant difference of epidermal cell width was observed between wild type and OsMADS25–OE lines (Fig 2C). These data suggest that *OsMADS25* might modulate cell length by maintaining ROS homeostasis.

### Overexpression of *OsMADS25* reduces the sensitivity to H_2_O_2_ by regulating the activities of ROS–scavenging enzymes

**Fig 3:**
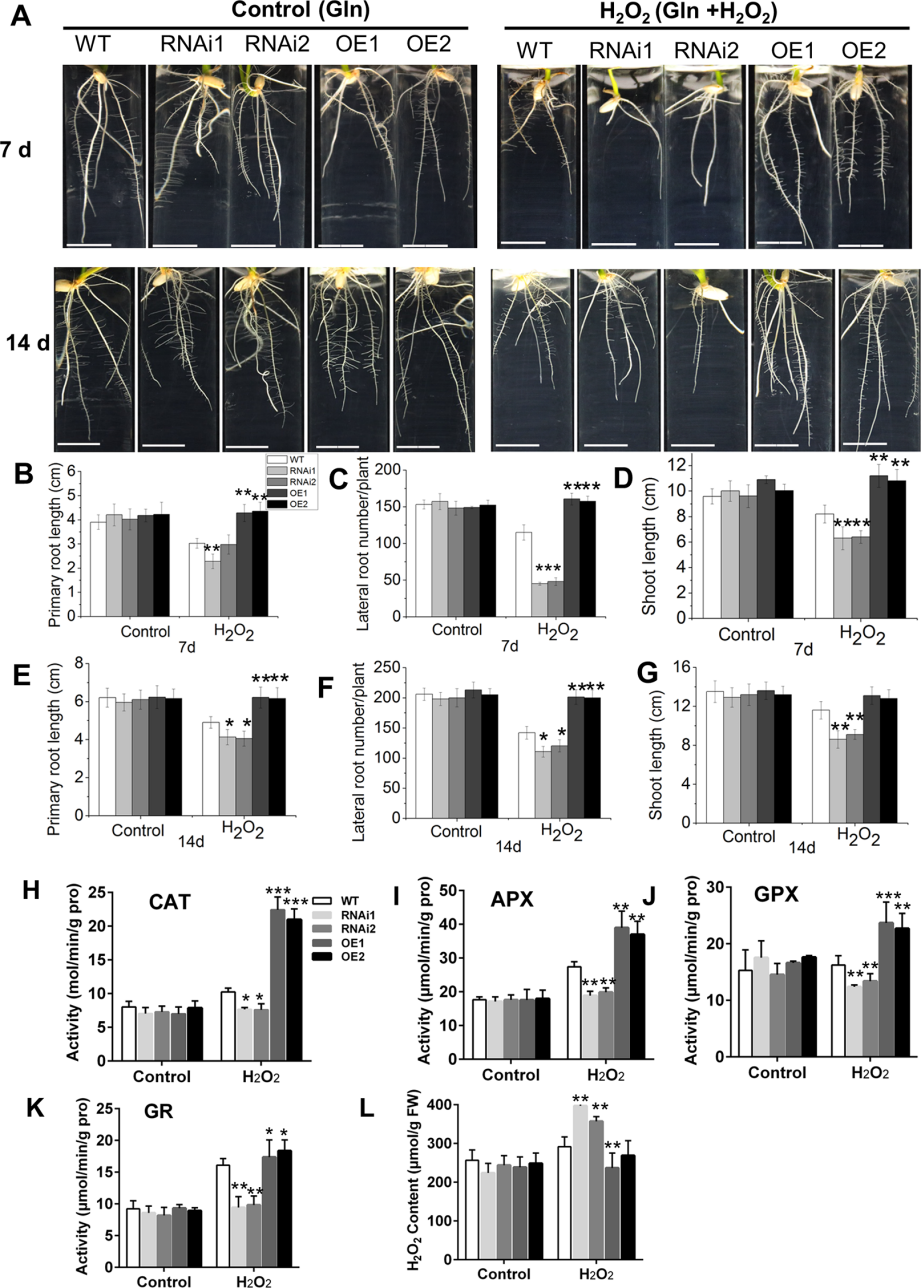
*OsMADS25* confers H_2_O_2_ tolerance by promoting ROS-scavenging capability. **A.** Root performance of wild type and *OsMADS25* transgenic seedlings in modified 1/2 MS medium (without nitrate, with 5 mM glutamine as the N nutrition) with or without 10 mM H_2_O_2_ for 7 or 14 days. Scale bars, 1 cm. **B–D.** Measurement of primary root length, lateral root number and shoot length of 7–day–old seedlings shown in image A. **E–G.** Measurement of primary root length, lateral root number and shoot length of 14–day–old seedlings shown in image A. **H–K.** Activities of antioxidant enzymes of CAT, APX, GPX and GR in roots of 7–day–old seedlings shown in image A. **L.** Quantification of H_2_O_2_ content in the roots of 7–day–old seedlings shown in image A. WT, wild type. RNAi1 and RNAi2, *OsMADS25*–RNAi transgenic lines. OE1 and OE2, *OsMADS25* overexpression lines. Three independent experiments were performed, and data are means ± SE (n = 15). The statistical significance of the measurements using one-way analysis of variance (ANOVA) was determined using Student’s *t*-test. Asterisks indicate the significant difference between *OsMADS25* transgenic lines and WT plants (^*^P < 0.05, ^**^P < 0.01 or ^***^P < 0.001).

Overexpression of *OsMADS25* reduced ROS accumulation in the roots, with a corresponding increase in cell elongation (Figs 1 and 2). Thus, the potential role of *OsMADS25* in maintaining ROS homeostasis was further evaluated by exposing the plants to H_2_O_2_. It has been reported that there is no significant difference in the root system growth between *OsMADS25* transgenic lines and wild type in modified 1/2 MS medium [with glutamine (Gln) as the N nutrition] [42]. Consistent with this, our results showed that the plant growth including PR elongation and LR branching was not affected by *OsMADS25* up–or down–regulation in modified 1/2 MS medium (S3A–S3D Fig). Further, we investigated the LRP density and epidermal cell size of PR, and no remarkable difference was observed between *OsMADS25* transgenic plants and wild type (S3E–S3I Fig). Meanwhile, *OsMADS25* does not affect ROS accumulation in shoots, roots and LRPs (S4 Fig). Thus, the modified 1/2 MS medium was used for investigating the responses of *OsMADS25* transgenic lines to H_2_O_2_, to exclude the effect of NO_3_^–^ on the root development. As shown in Fig 3A–3C, in the absence of H_2_O_2_, the PR elongation and LR density between the transgenic lines and wild type were highly similar; however, in the presence of 10 mM H_2_O_2_ for 7 days, the root growth in OsMADS25–RNAi lines was impaired severely, with decreased PR elongation and LR formation, whereas not affected in OsMADS25–OE lines, compared to their counterparts under control conditions. After exposed to H_2_O_2_ for 14 days, OsMADS25–OE roots still exhibited superior growth status, but the root growth in OsMADS25–RNAi lines was significantly repressed, compared to wild type (Fig 3A, 3E and 3F). The shoot growth in OsMADS25–OE lines is also not affected by H_2_O_2_ treatment, while decreased remarkably in RNAi lines, compared to that under control conditions (Fig 3D and 3G). The tolerance to oxidative stress is closely linked to the ROS–scavenging capability. As shown in Fig 3H–3K, after exposed to H_2_O_2_, the activities of ROS–scavenging enzymes such as CAT, APX, GPX and GR were significantly enhanced in OsMADS25–OE roots, whereas not altered remarkably in OsMADS25–RNAi lines, compared to their counterparts under control conditions, which led to an increase of H_2_O_2_ content (Fig 3L). Indeed, when exposed to H_2_O_2_, the expression of some ROS producers as well as scavengers in transgenic lines was also altered. The transcript levels of *OsRbohs* such as *OsRbohF* and *OsRbohG* were significantly elevated in OsMADS25–RNAi, while reduced in OsMADS25*–*OE plants, compared to that in wild type in modified 1/2MS medium (S5A and S5B Fig). For ROS scavengers, the expression of *OsCATB* and *OsGST4* was significantly enhanced in OsMADS25–OE lines, whereas reduced remarkably in RNAi lines (S6A and S6B Fig).

*OsMADS25* overexpression also reduced the sensitivity to H_2_O_2_ in standard 1/2 MS, indicated by less inhibition on the shoot and root growth (S7A–S7E Fig), and had less ROS accumulation (S7F Fig), as a result of increased activities of ROS–scavenging enzymes (S7G–S7J Fig). The role of *OsMADS25* in response to H_2_O_2_ was further investigated during seed germination. Almost all of the seeds were germinated on day 5 in the absence of H_2_O_2_, although down–regulation of *OsMADS25* clearly retarded seed germination (S7K and S7L Fig). However, after exposed to H_2_O_2_, the germination rate was significantly reduced in OsMADS25–RNAi seeds, whereas increased in OsMADS25*–*OE seeds, compared to that in wild type (S7K and S7L Fig). Together, these observations strongly support that *OsMADS25* could reduce the sensitivity to H_2_O_2_ during seed germination and post–germination growth by maintaining ROS homeostasis in rice. In fact, the ROS–scavenging capability regulated by *OsMADS25* was also confirmed through transient expression in the leaf of *Nicotiana benthamiana* under salinity stress (S8 Fig). These results suggest that *OsMADS25* has the capability of maintaining ROS homeostasis.

### OsMADS25 binds with the CArG–box motif and directly activates the transcription of *OsGST4 in vivo*

**Fig 4:**
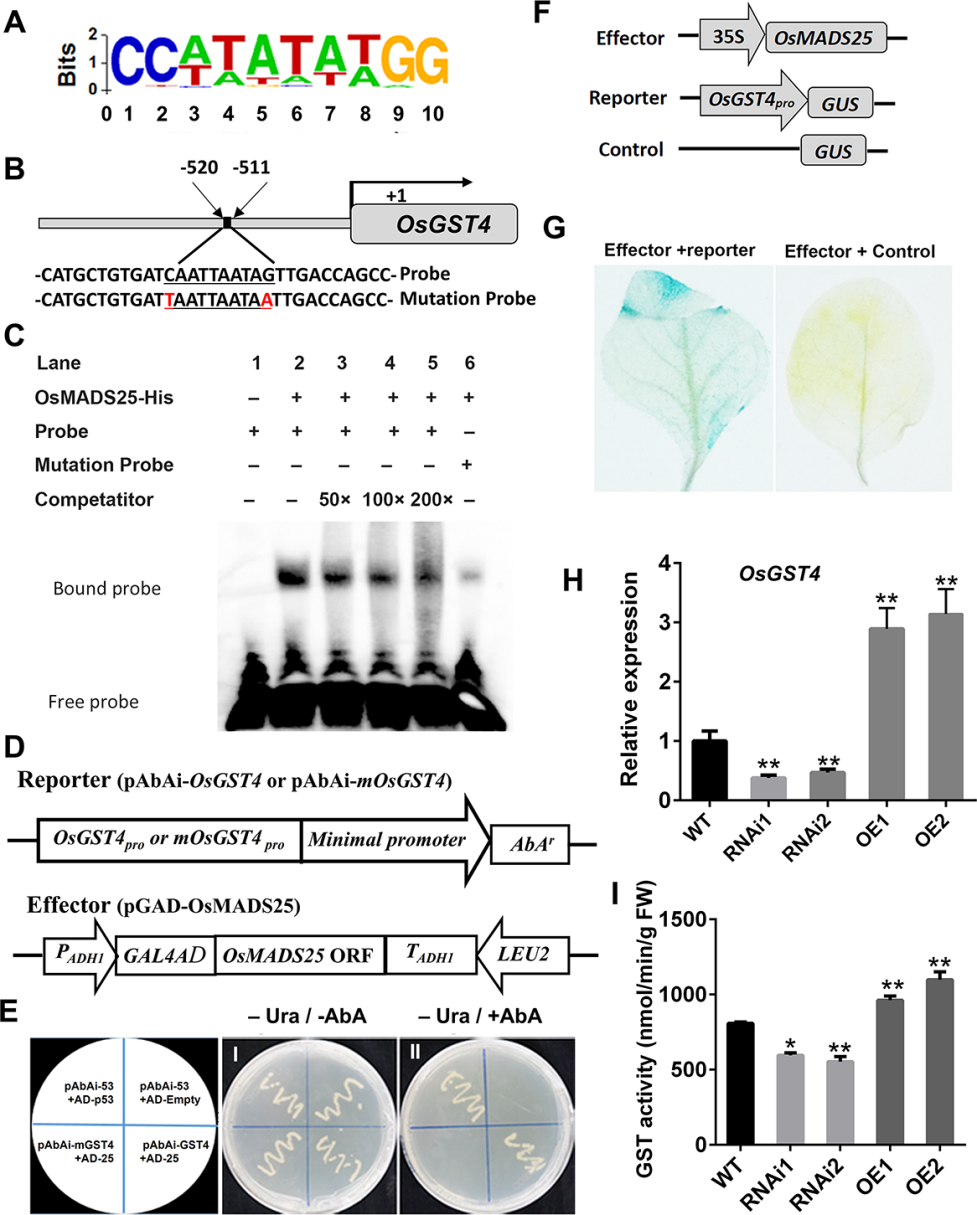
*Cis*–element binding ability and transcriptional–activation assays of OsMADS25. **A.** Nucleotide frequency distribution of the OsMADS25 core binding consensus sequence as determined ChIP–seq analysis. **B.** Schematic diagram of *OsGST4* promoter region showing the CArG–box motif. **C.** Electrophoretic mobility shift assays (EMSA) indicating OsMADS25 binding specific CArG–box motif located in the promoter region of *OsGST4*. **D.** Schematic diagrams of the effector and reporter used in the yeast one–hybrid assay. **E.** Transcriptional–activation assays showing OsMADS25 having transactivation activity in yeast. Panel (I) shows yeast cells containing distinct effector and reporter constructs grown on an SD/-Ura medium without AbA (–Ura; –AbA). 1. pGADT7–p53/p53–AbAi (positive control); 2. pGADT7/p53–AbAi; 3. pAbAi–mOsGST4/pGADT–OsMADS25; 4. pAbAi–OsGST4/pGADT–OsMADS25. Panel (II) shows that yeast cells shown in panel (I) cultured on SD/–Ura medium containing 200 ng ml^-1^ AbA (–Ura; +AbA). **F.** Schematic diagrams of the effector and reporter used for transient transactivation assay in *Nicotiana benthamiana*. **G.** Transactivation activity detected by GUS staining after reporter and effector plasmids coinfiltrated into the leaves of *N*. *benthamiana*. **H.** The transcript levels of *OsGST4* in 7–day–old wild type and *OsMADS25* transgenic roots by qPCR analysis. **I.** Measurement of GST activity in 7–day–old wild type and *OsMADS25* transgenic roots. WT, wild type. RNAi1 and RNAi2, *OsMADS25*–RNAi transgenic lines. OE1 and OE2, *OsMADS25* overexpression lines. Three independent experiments were performed. Data are means ± SE (n = 10). The statistical significance of the measurements using one-way analysis of variance (ANOVA) was determined using Student’s *t*-test. Asterisks indicate the significant difference between *OsMADS25* transgenic lines and WT plants (^*^P < 0.05, ^**^P < 0.01 or ^***^P < 0.001).

MADS–box proteins have been shown to regulate gene transcription by binding to a consensus core element CArG–box [45]. In our study, multiple ROS–scavengers were found to be potential targets regulated by OsMADS25 via ChIP–seq analysis (S2 Table). To further reveal the regulatory mechanism of *OsMADS25* in the tolerance to oxidative stress in rice, *OsGST4*, one of the potential targets of OsMADS25 was further analyzed (S2 Table and Fig 4A). We wondered whether OsMADS25 has the DNA binding activity on the promoter of *OsGST4*. Interestingly, *cis*–element scanning of the ~2kb promoter region of *OsGST4* showed one likely OsMADS25–binding CArG–box site at –511 to –520 bp from ATG position of *OsGST4* (Fig 4B). To confirm the binding specificity, we further performed EMSA using recombinant OsMADS25 and an oligonucleotide containing the CArG–box motif located in *OsGST4* promoter region. As shown in Fig 4C, a supershifted band was observed when labelled DNA probes containing the CArG–box motif was incubated with recombinant OsMADS25 (Lane 2), which means OsMADS25 was able to directly bind to the CArG–box motif. No supershifted signal was observed in the control sample containing only the labeled probe (Lane 1). Moreover, we observed decreased signals when a 50–, 100–, or 200–fold excess of unlabeled probe was added to the EMSA reaction as competitors (Lane 3–5,). Site mutations for the CArG–box motif were performed to further confirm the OsMADS25–binding specificity. When C and G in both ends of the CArG–box motif were mutated (mutation probe), OsMADS25 binding signal was decreased drastically (Lane 6).

To explore if OsMADS25 possesses transcriptional–activation activity, yeast one–hybrid assay was performed. The full–length cDNA of *OsMADS25* was fused in frame to *GAL4* activation domain in the pGADT7 vector, and the sequence of *OsGST4* promoter from –487 to –553 that harbours CArG–box motif was ligated into the pAbAi vector (Fig 4D). The yeast one–hybrid assay showed that OsMADS25 directly interacts with the sequence of *OsGST4* promoter (Fig 4E). To confirm that OsMADS25 can regulate the expression of *OsGST4 in vivo*, we performed transient transactivation assay in *N*. *benthamiana*. The sequence from –274 to –767 bp in *OsGST4* promoter was fused to the *GUS* reporter gene to use as the reporter, and *OsMADS25*, driven by the *CaMV35S* promoter, was used as the effector (Fig 4F). The reporter and effector plasmids were coinfiltrated into *N*. *benthamiana* leaves by *Agrobacterium*-mediated leaf disc infiltration. As we have expected, the *GUS* reporter gene was activated by coexpressing *OsMADS25* with the sequence of *OsGST4* promoter (Fig 4G). More importantly, the transcript abundance of *OsGST4* was found to be enhanced remarkably in OsMADS25–OE plants, but reduced greatly in RNAi lines (Fig 4H), and the GST activity in OsMADS25–OE plants was shown to be significantly higher, whereas lower in RNAi plants than that in wild type (Fig 4I). Taken together, these results indicate that OsMADS25 directly acts upstream of *OsGST4* and activates its transcription by interacting with the *cis*–element.

### *osgst4* mutant has reduced ROS–scavenging capability and tolerance to H_2_O_2_

**Fig 5:**
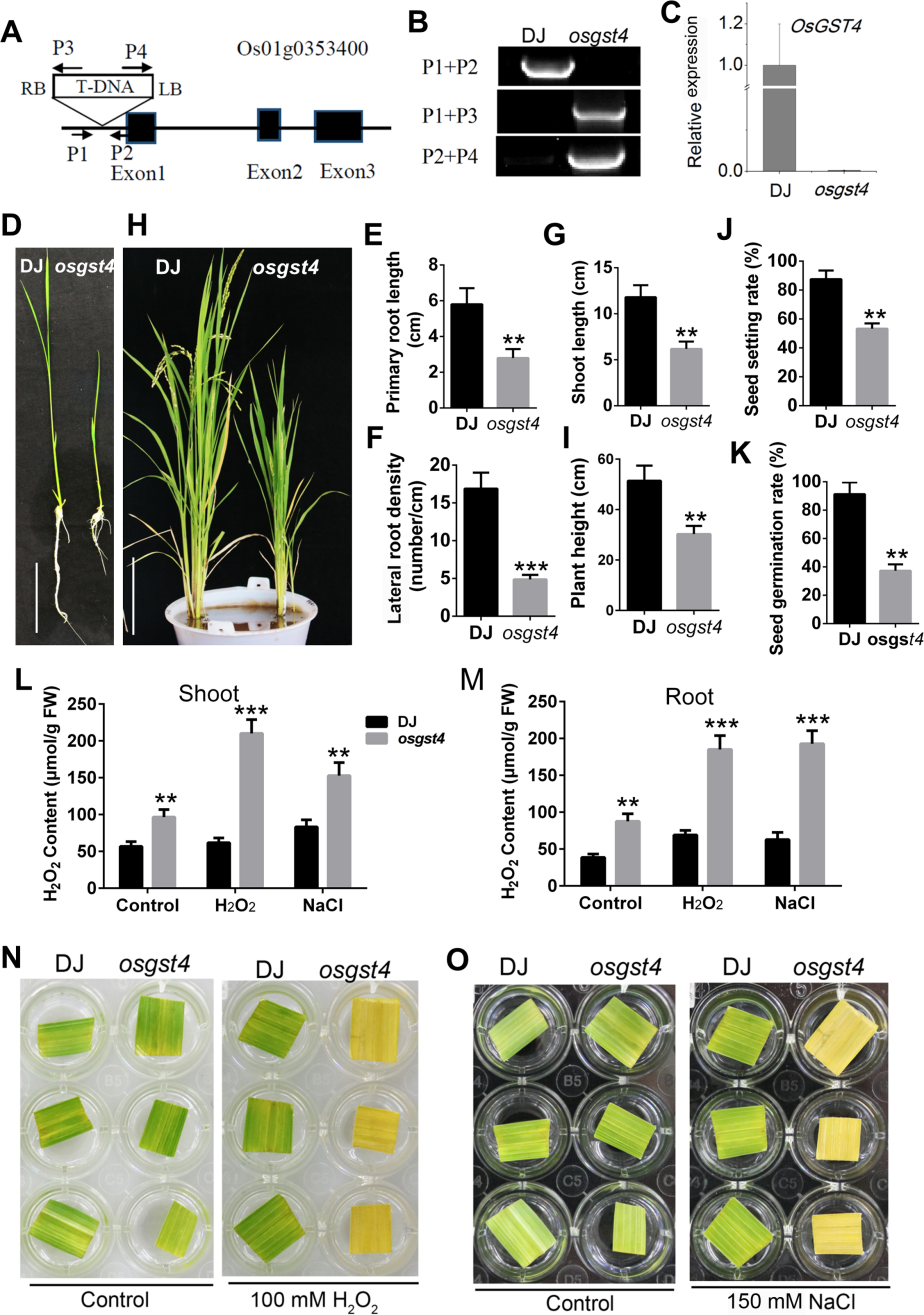
*osgst4* mutant exhibits defective growth and reduced tolerance to oxidative stress. **A.** Schematic diagram indicating the T–DNA insertion site in genomic region in *osgst4*. **B.** Genotyping of *osgst4* T_2_ seedlings performed via PCR analysis. **C.** Transcript levels of *OsGST4* in wild type (DJ) and *osgst4* mutant by qRT–PCR analysis. **D.** Seven–day–old seedlings of DJ and *osgst4* mutant grown in standard 1/2 MS medium. Scale bar, 4 cm. **E–G.** Measurement of primary root length, lateral root number and shoot length in image D. **H.** Plant architecture of DJ and *osgst4* mutant at mature stage. Scale bar, 10 cm. **I–K.** Comparison of plant height, seed setting rate and germination rate, respectively, between DJ and *osgst4* mutant in image H. **L** and **M.** Quantification of H_2_O_2_ content in the shoot and root of 7–day–old seedlings in response to H_2_O_2_ or NaCl, respectively. **N** and **O.** Detached leaves from 4–week–old DJ and *osgst4* plants exposed to 100 mM H_2_O_2_ or 150 mM NaCl for 3 days to indicate the oxidative stress tolerance. NBT, nitroblue tetrazolium. DAB, 3, 3’–diaminobenzidine. Three independent experiments were performed. Data are means ± SE (n = 30). The statistical significance of the measurements using one-way analysis of variance (ANOVA) was determined using Student’s *t*-test. Asterisks indicate the significant difference between *OsMADS25* transgenic lines and WT plants (^*^P < 0.05, ^**^P < 0.01 or ^***^P < 0.001).

Having elucidated OsMADS25 modulating root growth by regulating ROS scavenging via binding to the promoter region of *OsGST4*, we further provided convincing evidence to support a positive role of *OsGST4* in ROS scavenging in rice. A loss–of–function rice mutant that harbors a T–DNA insertion in *OsGST4* was obtained (Fig 5A and 5B). *OsGST4* contains three exons and two introns and is 5180 bp in total length, and the T–DNA insertion position is –417 bp from ATG, which caused the deletion of a 16–bp fragment (Figs 5A and S9A). Scarce transcripts of *OsGST4* were detected in *osgst4* by qPCR analysis (Fig 5C).

The contribution to plant growth and oxidative stress tolerance made by *OsGST4* was evaluated. *OsGST4* is expressed in various tissues (S9B Fig). As shown in Fig 5D–5G, the growth of *osgst4* was remarkably inhibited, and the PR elongation, LR formation and shoot growth were significantly reduced in 7–day–old seedlings in standard 1/2 MS medium, compared to wild type (DJ). At maturity, mutation of *OsGST4* affected plant growth severely, and the plant height was reduced over 40% in *osgst4*, compared to the wild–type counterpart (Fig 5H and 5I). It is notable that the seed setting rate in *osgst4* was also drastically reduced, with much higher blight grain rate and lower seed germination rate than wild type (Figs 5J, 5K and S9C). We then detected the ROS levels in *osgst4*. When exposed to H_2_O_2_ or NaCl, *osgst4* accumulated significantly higher levels of ROS than wild type (Fig 5L and 5M). Moreover, the response to oxidative stress showed that *osgst4* exhibited more sensitivity to H_2_O_2_ or NaCl, and the leaf chlorosis in *osgst4* was remarkably much quicker than that in wild type (Fig 5N and 5O), as a result of higher levels of ROS accumulation. As a strong support for this evidence, recombinant OsGST4 was shown to have the ROS–scavenging capability *in vitro* and improve the tolerance to oxidative stress in *Escherichia coli* (S9D Fig). These results suggest that the growth impairment and hypersensitivity to oxidative stress observed in *osgst4* may result from the enhancement of ROS levels, due to reduction of ROS–scavenging capability by *OsGST4* knockout.

### Overexpression of *OsMADS25* enhances the salinity tolerance in rice

**Fig 6:**
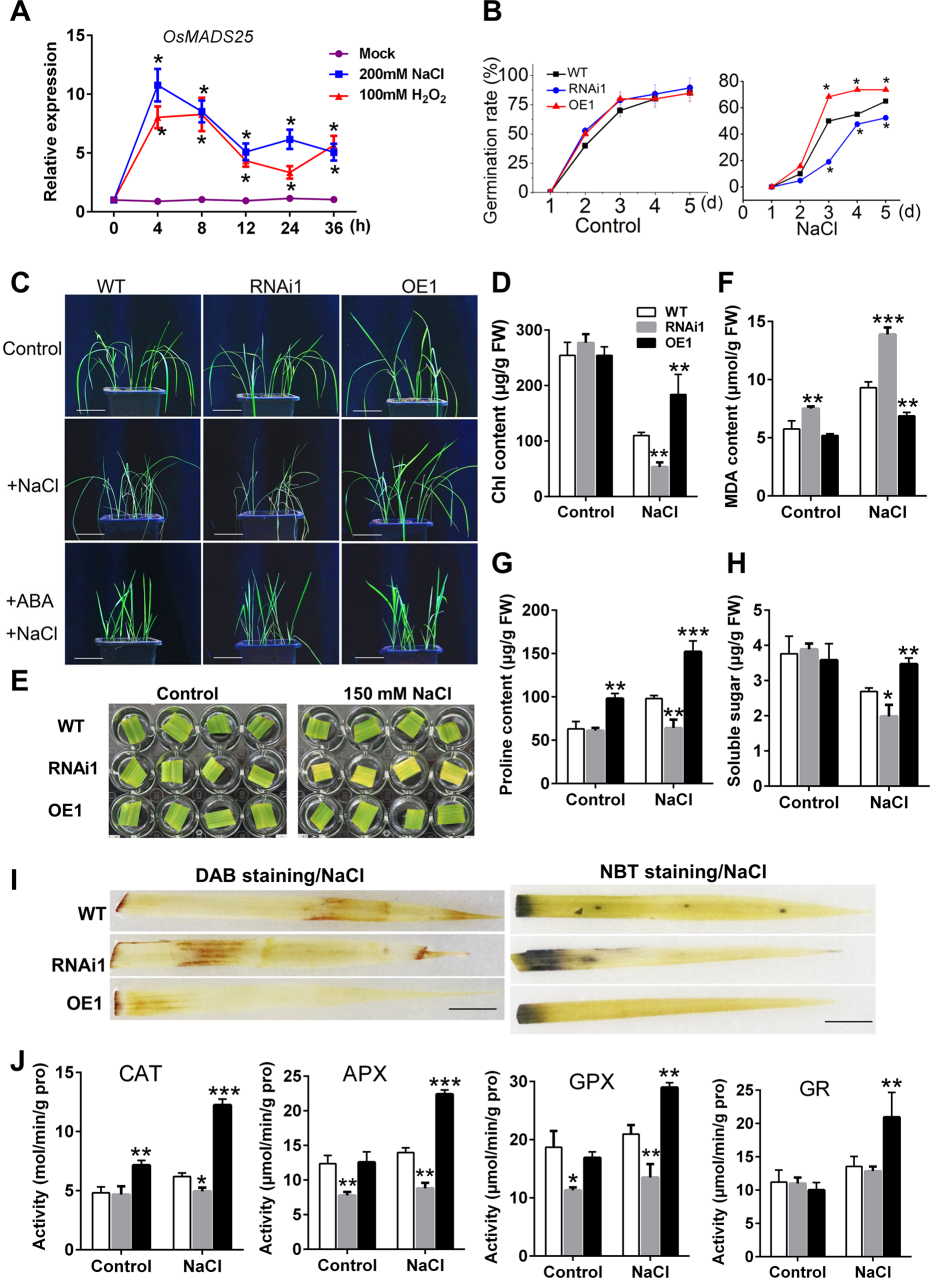
*OsMADS25* contributes to the salinity tolerance of rice. **A.** Time course of *OsMADS25* transcription induced by 100mM H_2_O_2_ and 150 mM NaCl via qPCR analysis. **B.** Comparison of seed germination rate between wild type and *OsMADS25* transgenic lines in the presence of 150 mM NaCl. **C.** Phenotype of wild type and *OsMADS25* transgenic seedlings exposed to salinity stress for 7 days. Scale bars, 5 cm. **D.** Measurement of chlorophyll content in wild type and *OsMADS25* transgenic seedlings exposed to salinity stress for 7 days. **E.** The detached leaves exposed to 150 mM NaCl for 3 days to indicate the salinity tolerance of wild type and *OsMADS25* transgenic lines. **F–H.** Measurement of the content of MDA, proline and soluble sugar in wild type and *OsMADS25* transgenic seedlings exposed to salinity stress for 7 days. **I.** DAB and NBT staining for the leaves from wild type and *OsMADS25* transgenic seedlings exposed to salinity stress for 7 days, respectively, to indicate ROS levels. Scale bars, 2 cm. **J.** Activities of ROS–scavenging enzymes CAT, APX, GPX and GR in wild type and *OsMADS25* transgenic roots exposed to salinity stress for 7 days. WT, wild type. RNAi1 and RNAi2, *OsMADS25*–RNAi transgenic lines. OE1 and OE2 *OsMADS25* overexpression transgenic lines. NBT, nitroblue tetrazolium. DAB, 3, 3’–diaminobenzidine. Three independent experiments were performed, and data are means ± SE (n = 15). The statistical significance of the measurements using one-way analysis of variance (ANOVA) was determined using Student’s *t*–test. Asterisks indicate the significant difference between *OsMADS25* transgenic lines and WT plants (^*^P < 0.05, ^**^P < 0.01 or ^***^P < 0.001).

Plants overexpressing–*OsMADS25* reduce the sensitivity to H_2_O_2_ (Fig 3), and the transcript levels of *OsMADS25* were remarkably enhanced in response to H_2_O_2_ and NaCl (Fig 6A). Thus, the function of *OsMADS25* in response to NaCl was explored during seed germination and post–germination growth. There was no significant difference in seed germination between wild type and *OsMADS25* transgenic lines in the absence of NaCl (Figs 6B and S10A). After exposed to 150 mM NaCl, seed germination in OsMADS25–RNAi lines was significantly inhibited, with only about 45% seeds germinated on day 5. Whereas, the inhibition of seed germination in OsMADS25–OE lines by NaCl is not severely, and 78% of seeds germination rate was observed, compared to 53% for wild type (Figs 6B and S10A). As to post–germination growth, wild type and *OsMADS25* transgenic plants grew normally with the similar chlorophyll levels in normal condition (Fig 6C and 6D). However, when exposed to salinity, the signs of stress were much severer in OsMADS25–RNAi lines, which exhibited serious chlorosis and wilting of the leaves, whereas the chlorosis of the OsMADS25–OE leaves was more delayed than that of wild type (Fig 6C). The total chlorophyll content, which reflects the presence of chlorosis, was reduced by 30% in the OsMADS25–OE lines, but 49% and 72% in wild–type and RNAi plants, respectively, compared with that in the untreated plants (Fig 6D). Consistent with the salinity–sensitive phenotype, the detached leaves of OsMADS25–RNAi lines showed a quicker rate of bleaching than that of wild type (Fig 6E). Under salinity conditions, MDA content, which reflects membrane injury and lipid peroxidation, was significantly elevated in OsMADS25–RNAi lines, whereas affected less severely in OsMADS25–OE lines in comparison with that in untreated plants (Fig 6F). There is also increasing evidence that plants accumulate proline and osmotic solutes to regulate their osmotic potential during salinity stress, which are important for protecting cells against increased levels of ROS under stress [46,47]. As shown in Fig 6G, although salinity stress greatly promoted the proline content in the leaves, OsMADS25–RNAi lines and wild type accumulated less proline than OsMADS25–OE plants, respectively. Under normal conditions, there was no significant difference in accumulation of soluble sugars between transgenic lines and wild type; however, under salinity stress, the content of soluble sugars in RNAi lines was remarkably reduced, whereas not changed significantly in OsMADS25–OE lines in comparison with that in wild type (Fig 6H). These results suggest that *OsMADS25* overexpression significantly improves salinity tolerance during seed germination and seedling growth in rice.

The abiotic stress led to ROS accumulation and ROS–associated oxidative injury. Accumulation of ROS in *OsMADS25* transgenic plants treated by NaCl was then determined by DAB and NBT staining, respectively. After the seedlings were grown in 150 mM NaCl for 3 days, weak staining by DAB and NTB was observed in the leaves of OsMADS25–OE plants, compared to that in wild type (Fig 6I), which is in accordance with their performance under salinity stress. The tolerance to salinity stress closely links to the activities of ROS–scavenging enzymes [10]. As shown in Fig 6J, after exposed to salinity stress, the activities of antioxidant enzymes in OsMADS25–OE lines were significantly enhanced, whereas not changed remarkably in wild type and RNAi lines, compared to their counterparts in untreated plants (Fig 6J). It is well recognized that ABA promotes stomatal closure to avoid water loss under various drought and salt stresses. Delayed wilting of the leaves observed in OsMADS25–OE lines in the presence of salinity prompted us to check the ratio of open stomata in response to exogenous ABA. As shown in S10B and S10C Fig, after exposed to exogenous ABA, compared to wild type, the ratio of open stomata in OsMADS25–OE leaves was significantly reduced, which suggests that overexpression of OsMADS25 might result in slower water loss in leaves. Together, these data indicate that overexpression of *OsMADS25* could enhance the ability of adaption to salinity stress by regulating the cellular ROS levels.

### Overexpression of *OsMADS25* improves root system development under salinity stress

To further evaluate the role of *OsMADS25* on root development under salinity stress, germinated seeds were grown in modified 1/2 MS medium including 150 mM NaCl. The root growth were not significantly affected by *OsMADS25* up–or down–regulation under control conditions (S11A–S11C Fig). However, after exposed to 150 mM NaCl for 7 days, the impairment of root growth in RNAi plants was much severer than that of wild type, whereas OsMADS25–OE plants showed less repression in PR length and LR density than wild type (S11A–S11C Fig). Corresponding to the root growth, the shoot growth in RNAi plants was also inhibited remarkably, leading to a significant decrease in the dry weight per plant (S11D and S11E Fig). Further we detected the activities of ROS–scavenging enzymes as well as ROS accumulation after exposed to NaCl. The activities of antioxidant enzymes in OsMADS25–OE lines were significantly increased, whereas not altered remarkably in wild type and RNAi lines, compared to that in untreated plants (S11F–S11I Fig). Accordingly, higher levels of ROS were found to be deposited in RNAi plants, while OsMADS25–OE line accumulated less ROS than wild type (S11J and S11K Fig). In parallel to ROS accumulation, the transcription of ROS-producers and ROS-scavengers was observed to change greatly (S5C and S6C Figs). Indeed, when grown in standard 1/2 MS medium including 150 mM NaCl, the root and shoot growth inhibition in OsMADS25–OE plants was also less severe than that in wild type (S12A–S12C Fig). Meanwhile, OsMADS25–OE lines had higher activities and expression levels of ROS-scavengers (S12D–S12H Fig), whereas had lower expression of ROS-producers than wild type (S12I Fig), after exposed to salt stress. Together, our findings suggest that OsMADS25 is an important regulator in improving plant root adaption to salinity stress by regulating the ROS levels.

### *OsMADS25* elevates ABA sensitivity and mediates ABA response in rice

**Fig 7:**
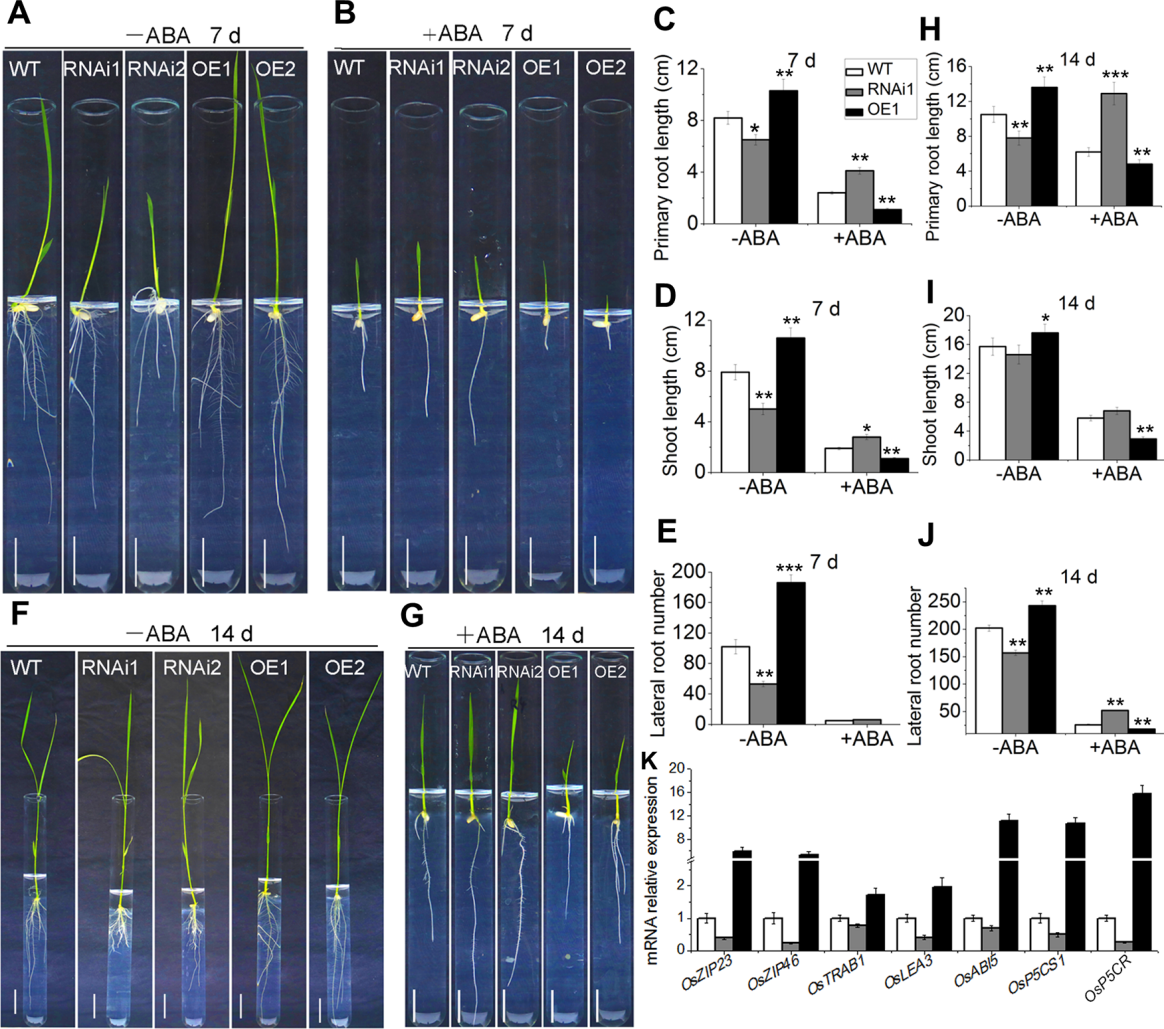
*OsMADS25* increases the ABA sensitivity of rice. **A and B.** Phenotype of wild type and *OsMADS25* transgenic seedlings grown in standard 1/2 MS medium with or without 5 μM ABA for 7 days. Scale bars, 2 cm. **C–E.** Statistical analysis of primary root length, shoot length and lateral root number of wild type and *OsMADS25* transgenic seedlings, respectively, in images A and B. **F and G.** Phenotype of wild type and *OsMADS25* transgenic seedlings grown in standard 1/2 MS medium with or without 5 μM ABA for 14 days. Scale bars, 2 cm. **H–J.** Statistical analysis of primary root length, shoot length and lateral root number of wild type and *OsMADS25* transgenic seedlings, respectively, in images F and G. **K.** Relative transcription levels of key genes involved in ABA*–*dependent stress response pathway in 2*–*week*–*old wild type and *OsMADS25* transgenic seedlings. To investigate the effect of ABA on rice growth, germinated seeds were grown in standard 1/2 MS medium with or without 5 μM ABA. WT, wild type. RNAi1 and RNAi2, *OsMADS25*–RNAi transgenic lines. OE1 and OE2, *OsMADS25* overexpression transgenic lines. Three independent experiments were performed, and data are means ± SE (n = 15). The statistical significance of the measurements using one-way analysis of variance (ANOVA) was determined using Student’s *t*–test. Asterisks indicate the significant difference between *OsMADS25* transgenic lines and WT plants (^*^P < 0.05, ^**^P < 0.01 or ^***^P < 0.001).

The phytohormone ABA serves as an endogenous messenger during stress responses in plants [26]. In order to determine whether *OsMADS25* is involved in the plant response via the ABA-dependent regulatory pathway, the biological function of *OsMADS25* in the response of post–germination growth to ABA was evaluated. In the absence of exogenous ABA for 7 days in standard 1/2 MS medium, OsMADS25–OE lines exhibited superior growth status, whereas the growth of RNAi lines was restrained, in comparison with wild type (Fig 7A and 7C–7E). Interestingly, in the medium supplemented with 5 μM ABA for 7 days, the growth of OsMADS25–OE seedlings was inhibited more severely than wild type, with remarkably reduced root and shoot growth (Fig 7B–7E), which suggests that the growth of OsMADS25–OE lines is arrested by ABA; by contrast, OsMADS25–RNAi lines exhibited less sensitivity to ABA than OsMADS25–OE plants, with longer PR and shoot than wild type (Fig 7B–7E). In ABA for 14 days, OsMADS25–OE plants were still more sensitive to ABA than wild type and OsMADS25–RNAi plants, especially the shoot growth (Fig 7F–7J). In addition, when seeds were geminated in standard 1/2 MS medium with 5 μM ABA, the germinative–growth in OsMADS25–OE lines was also strongly repressed, whereas RNAi lines were less sensitive, compared to wild type (S13A–S13C Fig). Together, these results indicate that *OsMADS25* increases the sensitivity to ABA during germinative–growth and post–germination growth, implying that *OsMADS25* might play an important role in ABA response.

To elucidate the role of *OsMADS25* in regulation of salinity tolerance in ABA–dependent pathway, the transcript levels of ABA–dependent stress–responsive genes *OsZIP23*, *OsZIP46*, *OsTRAB1*, *OsLEA3*, *OsABI5*, *OsP5CS1* and *OsP5CR* were investigated. Notably, the transcription of these genes were shown to be up–regulated in OsMADS25–OE lines under salinity stress (Fig 7K), which suggests that *OsMADS25* might be a transcriptional activator of these abiotic–stress associated genes in the ABA–mediated regulatory pathway; thus, up–or down–regulation of *OsMADS25* altered the expression of the set of genes.

ABA elevates ROS production by regulating the plasma membrane NADPH oxidases RBOHD and RBOHF [32]. We wonder whether exogenous ABA also affects ROS levels in *OsMADS25* transgenic lines. The result showed that, without ABA, higher levels of ROS were accumulated in OsMADS25–RNAi roots in standard 1/2 MS medium, whereas less ROS in OsMADS25–OE roots was detected than that in wild type (S13D and S13E Fig). By contrast, with 5 μM ABA treatment for 3 days, NBT and DAB staining was clearly weakened in the roots of OsMADS25–RNAi lines, but OsMADS25–OE lines exhibited the strongest staining (S13D and S13E Fig). Moreover, after a 3–d continuous ABA treatment, significantly more ROS had accumulated in the leaves of OsMADS25–OE lines in standard 1/2 MS medium, but less ROS was detected in OsMADS25–RNAi lines than that of wild type (S13F–S13G Fig), which suggests that exogenous ABA reduces ROS accumulation in RNAi lines, but enhances ROS levels in OsMADS25–OE lines. Along with the fact that OsMADS25–OE roots are more sensitive to ABA (Fig 7), it could be proposed that, ROS may function in the downstream of ABA–mediated regulatory pathway in regulating root elongation, and the enhancement of ROS–scavenging capability by *OsMADS25* may be, at least partially, mediated by ABA–mediated regulatory pathway.

### OsMADS25 directly activates the transcription of *OsP5CR*

**Fig 8:**
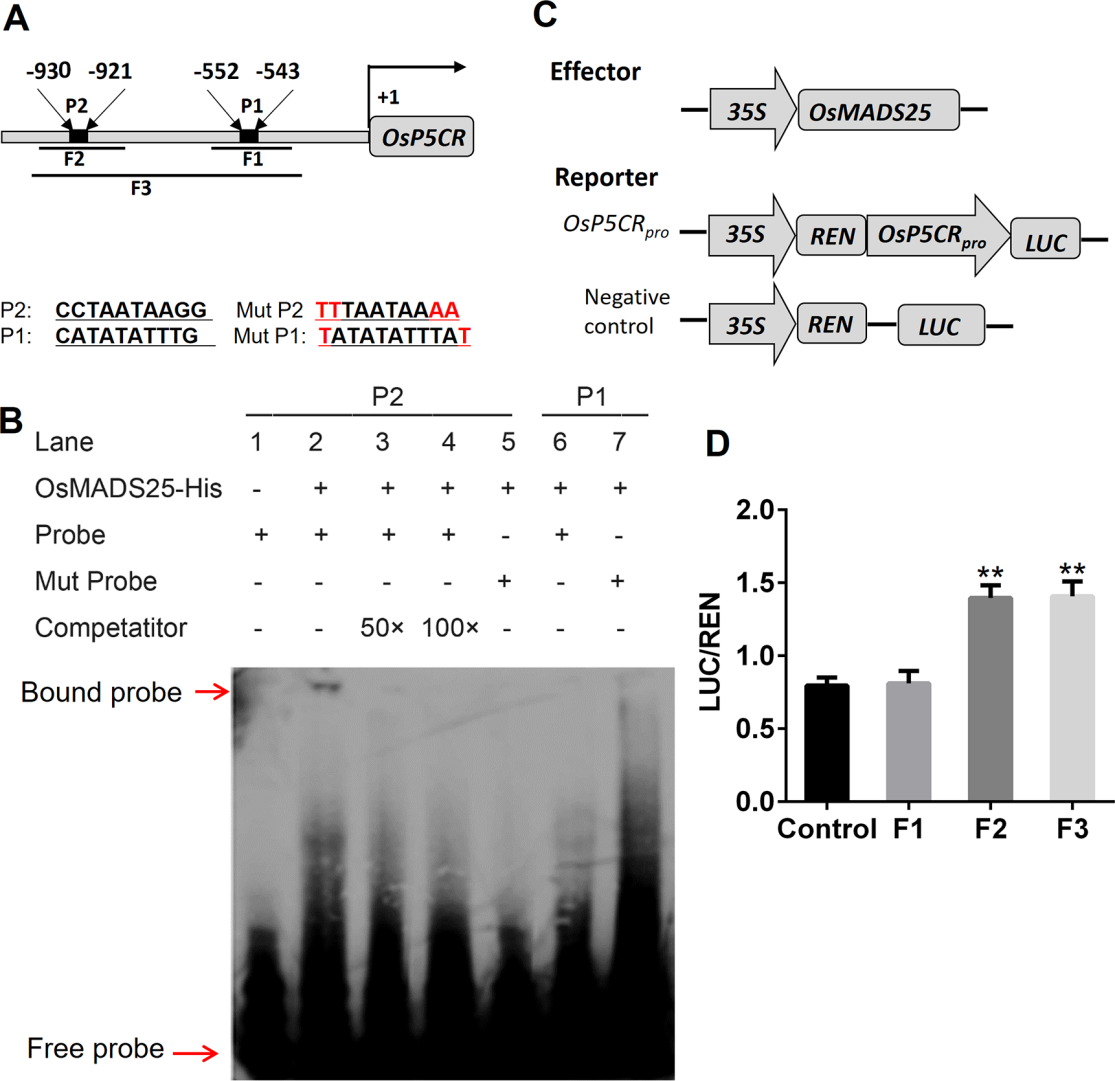
OsMADS25 is a transcriptional activator of *OsP5CR*. **A.** Schematic diagram of *OsP5CR* promoter region showing the CArG–box motifs. **B.** Electrophoretic mobility shift assays (EMSA) indicating OsMADS25 binding specific CArG–box motifs. **C.** Schematic diagrams of the effector and reporter used for transient transactivation assays in in rice protoplasts. *REN*, *Renilla luciferase*; *LUC*, firefly luciferase. **D.** Transactivation activity reflected by LUC activity of LUC/REN ratio. Data are means ± SE (n = 6). Probes P1 and P2 indicated oligonucleotides used for EMSA. Fragments F1, F2 and F3 indicated DNA fragments used for transcriptional–activation assays. The statistical significance of the measurements using one-way analysis of variance (ANOVA) was determined using Student’s *t*–test. Asterisks indicate the significant difference between treatment and control (^*^P < 0.05, ^**^P < 0.01 or ^***^P < 0.001).

OsMADS25–OE plants exhibited increased tolerance to salinity and accumulated much higher levels of proline than wild type (Fig 6). The transcripts of *OsP5CR*, responsible for proline biosynthesis, were also shown to be more abundant in OsMADS25–OE plants than that in wild type, whereas significantly reduced in OsMADS25–RNAi lines, in response to salinity (Fig 7K). Moreover, ChIP–seq analysis showed that *OsP5CR* is a candidate target regulated by OsMADS25 (S2 Table). Then, EMSA was performed to test whether OsMADS25 has DNA binding specificity for the *OsP5CR* promoter with two CArG–box motifs (Fig 8A). As shown in Fig 8B, a shifted band was clearly detected when probe 2 (P2) in the *OsP5CR* promoter region was incubated with OsMADS25 protein (lane 2); by contrast, no shifted bands were observed when the probe 1 (P1) were incubated with recombinant OsMADS25 (lane 6). Competition assay and probe mutation confirmed that this binding was specific (lane 3–5). We then explored whether OsMADS25 directly regulates *OsP5CR* expression *in vivo*. The firefly luciferase reporter driven by *OsP5CR* promoter (*OsP5CR*_*Pro*_::*LUC*) and *Renilla* luciferase driven by 35S promoter (*35S*::*REN*; as an internal control) were constructed in the same plasmid (Fig 8C). After transiently expressed in rice protoplasts, the LUC and REN activities were then measured and the LUC activity was normalized to REN activity. As shown in Fig 8D, coexpression of *35S*::*OsMADS25* with *OsP5CR*_*Pro*_::*LUC* increased the LUC/REN ratio, and LUC activity was approximately 1.75–fold of that in the control (Fig 8D). Thus, our data indicate that OsMADS25 is an upstream transcriptional regulator of *OsP5CR*. This result suggests that *OsP5CR* expression activated by OsMADS25 directly might be involved in the response to salinity.

### *OsMADS25* might be involved in auxin signaling to regulate root system architecture

Auxin plays critical roles in the root growth and development, and ROS have been proposed to act as important signals during auxin–regulated LR initiation and emergence [48–50]. There is significant difference in the root system development between *OsMADS25* transgenic lines and wild type (Fig 1), which suggests that up–or down–regulation of *OsMADS25* might alter the auxin signaling. *OsYUCCA4* (*OsYUC4*), responsible for auxin biosynthesis, was found to be the potential target of OsMADS25 through ChIP–seq analysis (S2 Table). Knock down of *OsYUC4* seems to impair plant growth in rice [51]. We wonder whether *OsMADS25* regulates the transcription of *OsYUC4 in vivo*, and then the LUC activity was measured by transient coexpression of *OsYUC4*_*Pro*_::*LUC* and *35S*::*OsMADS25* in rice protoplasts (S14A Fig). LUC activity in protoplasts transformed with *LUC* under the control of the *OsYUC4* promoter was 2.76–fold of that of the control (S14B Fig), which suggests that *OsMADS25* might elevate auxin biosynthesis by activating *OsYUC4*. We then detected the transcript levels of genes involved in auxin biosynthesis and signaling. As shown in S14C Fig, the expression of *OsYUC4*, *OsARF1* and *OsARF16* was increased significantly in OsMADS25–OE lines, while reduced in OsMADS25–RNAi lines. However, for *OsIAA14*, the mRNA levels were remarkably reduced in OsMADS25–OE lines but enhanced in RNAi lines (S14C Fig), which suggests that the overexpression of *OsMADS25* might promote the auxin signaling. When plants are exposed to environmental stresses, stress-induced accumulation of ROS might alter auxin signaling [52–54]. In combination of induced expression of *OsMADS25* by NaCl and H_2_O_2_ (Fig 6A), it is supposed that auxin signaling promoted by OsMADS25 might a positive regulator of stress tolerance.

## Discussion

### *OsMADS25* confers salinity tolerance by enhancing antioxidation capacity and proline biosynthesis

Numerous studies have revealed the involvement of ROS–scavenging capacity in plant tolerance to salinity stress [10,55]. In accordance with these above findings, OsMADS25–OE plants showed increased tolerance to salinity and oxidative stress, with a corresponding increase in the activities of antioxidant enzymes and a decrease in ROS accumulation (Figs 3 and 6 and S7 and S8). These results showed that the functions of *OsMADS25* in salinity tolerance might be associated with the regulation of antioxidation ability. GSTs are thought to play an important role during oxidative stress in plant [56]. Up–regulation of *PjGSTU1* from *Prosopis juliflora* in tobacco confers drought tolerance and recombinant PjGSTU1 possesses GST and GPX activities [57]. In another report, mutation of *Arabidopsis GSTU17* affected the accumulation of GSH and ABA, and *gstu17* exhibited ABA hypersensitivity during seed germination [58]. In our study, *OsMADS25* elevated the activities of several ROS–scavenging enzymes under oxidative stress (Figs 3, 6, S7, S8, S11 and S12). As the strong evidence supporting *OsMADS25* modulating ROS levels in rice, OsMADS25 acts upstream of *OsGST4* and positively regulates its expression *in vivo* (Fig 4). Moreover, recombinant OsGST4 has the ROS–scavenging capability *in vitro* (S9D Fig). It is notable that the detached leaves of *osgst4* mutant also exhibited much higher sensitivity to oxidative and salinity stresses (Fig 5N and 5O). These results suggest that the elevated activities of ROS–scavenging enzymes significantly contribute to lower ROS accumulation and less oxidative damage in OsMADS25–OE lines, which is associated with the improved tolerance to salinity stress.

The osmoprotective function of proline has been well known under environmental stresses, although the correlation between proline accumulation and abiotic stress tolerance in plants is not always apparent. Osmotic stresses activates proline biosynthesis, which is controlled by the *P5CS* and *OsP5CR* [18]. *Arabidopsis P5CS1* is induced by osmotic and salt stress and is activated by an ABA–dependent pathway and H_2_O_2_–derived signals [17,19]. Indeed, proline has been shown to protect and stabilize ROS–scavenging enzymes and activate alternative detoxification pathways. Recent research showed that the increased proline accumulation and salinity tolerance induced by ABA results from the up–regulation of *OsP5CR* in rice [59]. In accordance with these findings, in our study, altered ABA response and enhanced expression of ABA–dependent stress–responsive genes were observed in OsMADS25–OE lines, coupled with increased *OsP5CR* expression and proline accumulation (Figs 6G and 7K). These data suggest that ABA–dependent regulatory pathway is linked to proline biosynthesis controlled by *OsP5CR* in OsMADS25–OE lines.

### ROS, ABA and proline, interact with each other?

In this study, we showed that *OsMADS25* regulated root cell elongation by maintaining ROS homeostasis, and the perturbed balance of ROS led to an excess of ROS and a corresponding decrease in cell elongation in *OsMADS25*–RNAi plants (Figs 1 and 2). Corresponding to the alteration of ROS levels, altered ABA sensitivity was also observed (Figs 7 and S13), which suggests that ROS alteration is correlated to ABA sensitivity. It has been reported that alterations in ROS levels can affect ABA biosynthesis and signaling, as well as change ABA sensitivity [12,19,60], and ABA can also regulate the expression of ROS–producing and–scavenging genes [61,62]. For instance, recent research reveals a link between ABA signaling and H_2_O_2_ production via G–proteins that are shown to promote H_2_O_2_ production but negatively regulate ABA response [63,64]. These data suggest that there are likely to be different mechanisms by which ABA signaling and ROS production interact and regulate each other. All of these observations strengthen the link between the changed H_2_O_2_ levels and altered ABA response in *OsMADS25* transgenic plants (Figs 7 and S13). Previous reports demonstrated that salinity–induced proline accumulation is dependent on ABA, which is also consistent with the proposal that proline is associated with redox regulation and that might serve as an antioxidant [65,66]. In many cases, abitotic stress gives rise to various metabolic changes, known as elevated ROS levels [9]. Along with increased ROS, ABA signaling and ABA–dependent proline accumulation, have been proposed to be crucial components of cross tolerance to various stresses [67].

### The interplay between auxin and the ABA–mediated regulatory pathway?

A crosstalk between ABA and ROS signaling has been proposed and the role of ABA in adaptive abiotic stress responses via redox metabolism has been elucidated [10,29,30]. ABA is often recruited as the primary signal activating the transcription of stress–responsive genes [68], and ABA–triggered ROS production is involved in this process [8]. *OsMADS25* was induced by oxidative stress (Fig 6A), therefore, there is a reasonably strong case that the induction of *OsMADS25* expression operates via the ABA–mediated regulatory pathway. ABA triggers a signaling cascade that up–regulates a suite of abiotic stress–responsive genes [69,70], which were also shown to be up–regulated in OsMADS25–OE lines under salinity (Fig 7K). Intriguingly, *OsMADS25* expression was slightly inhibited by ABA (S15 Fig), which is consistent with previous research [71]. Overall, the decreased ratio of open stomata in response to ABA, the enhancement of ABA sensitivity and the transcription up–regulation of these genes in OsMADS25–OE lines (Figs 7 and S10B and S10C) provide firm evidence for a positive role of OsMADS25 in a stress–responsive ABA–mediated regulatory pathway. Thus, ABA enhances the transcription and activities of ROS network genes, and defects in this network can also disrupt the expression of stress–responsive ABA–dependent genes [33].

Crosstalk between ABA and auxin has been proposed that ABA-potentiating auxin-mediated growth repression is considered as the result of promoted auxin signaling or increased auxin flow and/or auxin levels by ABA [72]. For instance, the auxin signaling-defective mutants have reduced sensitivity to ABA in the root elongation [73,74]. Alternatively, auxin can also enhance ABA-mediated seed germination inhibition and leaf senescence under various oxidative stresses [75,76]. These studies highlight coenhancement between ABA and auxin signaling or auxin homeostasis during seed germination and seedling development [2,72]. In accordance with these data described above, in our study, overexpression of *OsMADS25* seemed to promote auxin signaling (S14 Fig), and OsMADS25–OE lines also showed increased sensitivity to exogenous ABA (Figs 7 and S13). As a fact, besides genes for antioxidant enzymes and proline biosynthesis, genes for auxin biosynthesis and auxin signaling as well as calcium signaling were potential targets regulated by OsMADS25, indicated as ChIP-seq analysis (S2 Table), which suggests that salinity tolerance conferred by OsMADS25 might be the result of crosstalk of multiple cell signaling pathways.

**Fig 9:**
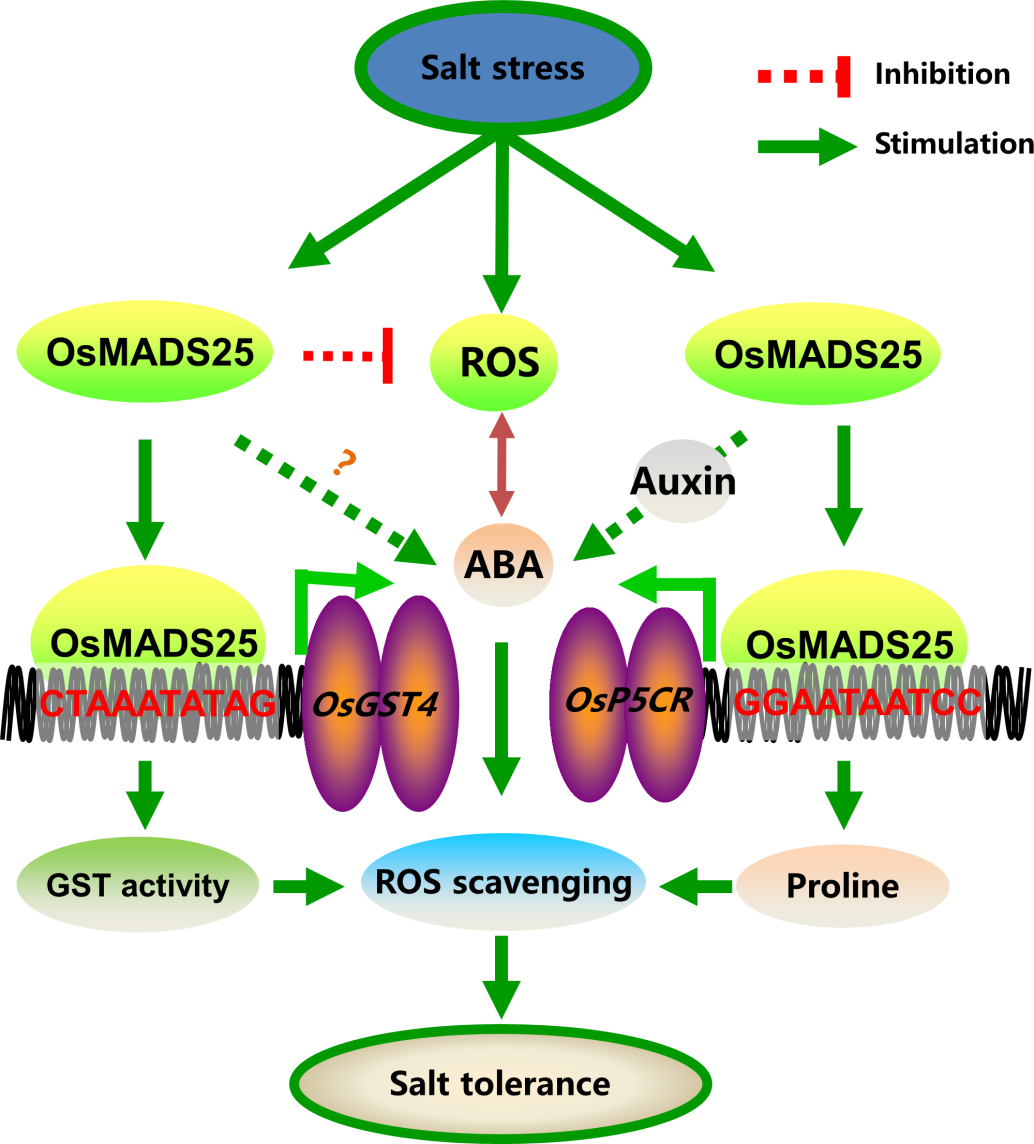
Proposed working model for *OsMADS25* in the regulation of growth and salinity tolerance in rice. Salinity stress produces ROS as well as induces *OsMADS25* expression. OsMADS25 directly activates the transcription of *OsGST4* and ABA–dependent *OsP5CR* to increase antioxidant responses and proline accumulation to fulfill ROS-scavenging, in combination with ABA–dependent abiotic stress–responsive regulatory pathway; besides, *OsMADS25* might regulate the root growth via auxin signaling, which also enhances ABA signaling in oxidative stress, thereby accounting for the positive role of *OsMADS25* in the growth and salinity tolerance in rice.

Based on our results, our overall proposition is that, salinity stress produces ROS as well as induces *OsMADS25* expression. OsMADS25 directly activates the transcription of *OsGST4* and ABA-dependent *OsP5CR* to increase antioxidant responses and proline accumulation, in combination with ABA–dependent abiotic stress–responsive regulatory pathway, to fulfill ROS-scavenging; besides, *OsMADS25* might regulate the root growth via auxin signaling, which enhances ABA signaling in oxidative stress, thereby explaining the positive role of *OsMADS25* in the root growth and salinity tolerance in rice (Fig 9).

## Materials and methods

### Plant materials and stress treatments

Rice (*Oryza sativa* L. ssp. japonica cv. Nipponbare) was used as wild type for physiological experiments and genetic transformation. The T–DNA insertion mutant line PFG_3A–11112.L (*osgst4*) in an *Oryza sativa* ssp *japonica* cv Dongjin (DJ) background was obtained from RiceGE (the Rice Functional Genomics Express Database) in Korea. Plants were grown in a greenhouse under a 14–h photoperiod (300 μmol photons m^−2^ s^−1^) at 30°C. For the phenotype analysis during post–germination growth, germinated seeds were grown on standard 1/2 MS medium or modified 1/2 MS medium (without nitrate, with 5 mM glutamine as the sole N nutrition). For salinity stress testing, seedlings were grown in modified 1/2 MS medium containing 150 mM NaCl, 10 mM H_2_O_2_ or 5 μM ABA. For oxidative stress of detached leaved, 100 mM H_2_O_2_ was used. For salinity stress in soil, germinated seeds were grown in pots (about 1 dm^3^) containing filled mixture of soil and vermiculite (1:1). After 7 days of growth, plants were irrigated every 3 days with 200 mL of 200 mM NaCl solution. The control plants were irrigated with 200 mL water every 3 days. For the seed germination or germinative–growth assays under stress, the seeds were placed on filter paper moistened with NaCl (150 mM), H_2_O_2_ (10 mM) or ABA (5 μM) solutions, and seed germination and germinative–growth in water as control. The germination rates were recorded daily.

### Vectors construction and plant genetic transformation

For overexpression vector, the ORF of *OsMADS25* was cloned into pCAMBIA1301, driven under the *35S* promoter. We used the pENTR/D–TOPO vector system to construct RNAi vector [77]. Genetic transformation of rice was performed using *Agrobacterium*–mediated methods [78]. Primers for DNA vectors construction and transformants screening are listed in S1 Table.

### Stomatal observation

Full expanded young leaves of 5 day-old rice seedlings with or without 50μM ABA treatment in MES–KCl buffer (50 mM KCl, 10 mM MES-KOH, pH = 6.15) for 2 h were used for stomatal observation. Stomatal closure was monitored by Hitachi SU3500 scanning electron microscope with a −40°C cool stage.

### ROS assays

We used nitroblue tetrazolium (NBT) staining to detect O_2_^–^ and 3, 3′–diaminobenzidine (DAB) staining for H_2_O_2_, as described previously [7]. H_2_O_2_ quantification was performed according to the method as described previously [79]. The hydrogen peroxide detection kit was used. Briefly, 10 mg plant tissue samples were snap frozen in liquid nitrogen and homogenized, and 200 μl lysate was added and centrifuged at 12,000 g at 4°C for 3–5 minutes. The supernatant was taken for subsequent measurement. After taking 50 μl supernatant to a 96-well plate, 100 μl of hydrogen peroxide detection reagent was added, and the reactive solution was mixed gently by vortexing and incubated for 30 minutes at room temperature. Then A560 wavelength was measured, and the hydrogen peroxide concentration was calculated from the standard curve.

### Measurement of chlorophyll, proline, soluble sugar, and malondialdehyde (MDA) content

Leaves from plants exposed to salinity stress for 7 days were used to measure chlorophyll, proline, soluble sugars and MDA levels, and plants grown in normal conditions were used as control. Total chlorophyll content was determined by the protocol as described previously [80]. Briefly, 100 mg of fresh leaves were homogenised in liquid nitrogen, and 5 ml of 80% acetone was added. After being incubated in dark for 1 h, the reaction mixture was centrifuged at 12,000 g for 3 min. The supernatant was used to measure the absorbance spectrophotometrically at 645 and 663 nm against 80% acetone as blank. The chlorophyll content was determined as follows: Total chlorophyll (μg/ml) = 20.2 (A_645_) + 8.02 (A_663_).

Free proline content was measured using the reported method [30]. Tissue samples of 1 g were pulverized in liquid nitrogen and homogenized in a 3% aqueous solution of sulfosalicylic acid. The samples were then centrifuged at 12,000 g for 15 minutes at 37°C and 2 ml of the supernatant was taken. An equal amount of acidic ninhydrin and glacial acetic acid were added, and the reaction mixture was kept in boiling water for 1 h. The reaction was stopped on ice, and 4 ml of toluene was added to the reaction mixture to collect the material separated from the aqueous phase. The absorbance of toluene was measured at 520 nm and the proline standard curve was used to calculate the proline content.

Soluble sugar content was determined by the anthrone method [81]. Briefly, after fresh leaf samples were homogenized with deionized water, the mixture was filtered and treated with 5% phenol and 98% sulfuric acid, and the absorbance at 485 nm was determined with a spectrophotometer.

MDA content was determined as previously described [81]. Approximately 0.1 g of leaf samples were ground in 10 ml of 10% trichloroacetic acid (TCA). The homogenate was centrifuged at 10,000 rpm for 20 min. The reaction mixture containing 2 ml of extract and 2 ml of TBA was heated at 95°C for 30 min, quickly cooled on ice, and then centrifuged again at 10,000 g for 20 min. The absorbances at 450, 532, and 600 nm were determined using an ultraviolet spectrophotometer.

### Antioxidant enzyme activity assays

Fresh leaf samples were used for enzyme extraction. All operations were carried out at 4°C. CAT activity was measured according to the method as described previously [82]. Decrease in absorbance of H_2_O_2_ was measured in 1 ml of reaction mixture containing 10 mM H_2_O_2_ and 20 μl of enzyme extract in 50 mM of K_3_PO_4_ buffer (pH = 7). Specific enzyme activity was expressed as l mole of H_2_O_2_ decomposed min^-1^ mg protein^-1^. The activities of APX and glutathione reductase (GR) were determined by the reported method [83]. The reaction mixture of 1 ml comprised of 0.5 mM ascorbate, 0.1 mM H_2_O_2_, 0.1 mM EDTA, K_3_PO_4_ buffer (pH = 7) and 10 μl of enzyme extract. Decrease in absorbance was observed spectrophotometrically at 290 nm at 25°C. One unit of enzyme activity was expressed as the amount of enzyme required to oxidise 1 μM of ascorbate minute^-1^ g tissue^-1^. The specific activity of GR is determined by analysing the decrease in absorbance at 340 nm. One ml reaction mixture used for the assay contained 50 mM K_3_PO_4_ buffer (pH = 7.8), 1 mM EDTA, 1 mM oxidized glutathione (GSSG) and 25 μl enzyme extract. The reaction was started by adding 0.1 mM NADPH. Enzyme activity was expressed as μmol of NADPH oxidised min^-1^ mg protein^-1^.

GST activity was determined according to the protocol as described previously [84]. The reaction mixture contained 100 mM Tris-HCl buffer (pH = 6.5), 1.5 mM GSH, 1 mM 1–chloro–2, 4–dinitrobenzene (CDNB), and enzyme extract in a final volume of 0.7 ml. The enzyme reaction was initiated by the addition of CDNB and the increase in absorbance was measured at 340 nm for 1 min. The activity was calculated using the extinction co-efficient of 9.6 mM^−1^ cm^−1^. GPX activity was investigated by measuring the increase in absorbance at 436 nm, by the method as described previously [85]. The reaction mixture was prepared in 50 mM K_3_PO_4_ buffer (pH = 7) with 9 mM guaiacol, 10 mM H_2_O_2_ and 33 μl of enzyme extract. The enzymatic activity of GPX is expressed as the amount of enzyme required to produce 1 μmol guaiacol dehydrogenation product min^-1^ mg protein^-1^.

### Propidium iodide (PI) staining procedures

For confocal microscopy analysis, root tips of 5–day–old seedlings were stained with PI at a concentration of 10 mg/ml for 5 minutes, and then washed three times with double–distilled water. After staining and mounting, all root tips were viewed using a Leica confocal laser microscope.

### RNA extraction, semi–quantitative and quantitative real–time PCR (qPCR) analysis

Total RNA was extracted using TRIzol reagent. Reverse transcription and qPCR analysis were performed as described previously [86]. The specific primers used for expression analysis are listed in S1 Table.

### Transcriptional activation assay in yeast

The Matchmaker Gold Yeast One–Hybrid Library Screening System (Clontech) was used to examine protein binding. A fragment from *OsGST4* promoter region containing the CArG–box motif was cloned into the pAbAi vector to act as the reporter. The reporter plasmid was introduced into the yeast strain Y1HGold, and the background *AbA*^r^ expression of the reporter strain was tested. The ORF of *OsMADS25* was fused in–frame with the *GAL4* activation domain of the one–hybrid vector pGADT7, forming pGAD–OsMADS25 as the effector. The reporter strain was transformed with pGADT7 or pGAD–OsMADS25. Primers for DNA vector construction are listed in S1 Table.

### Transient transactivation in *N*. *benthamiana* and dual–luciferase assay

We used pGreenII cloning vectors to construct transactivation vectors [87]. The ORF of *OsMADS25* was cloned into pGreenII 62–SK to act as the effector. For reporter construction, pGreenII 0800–LUC or pGreenII 0800–GUS was used. pGreenII 0800–GUS was generated from pGreenII 0800–LUC by substituting *LUC* with *GUS* in the restriction enzyme site of *Xba*I.

The promoter sequence of *OsGST4* was cloned into pGreenII 0800–GUS, acting as the reporter, and the empty vector pGreenII 0800–GUS acts as the control. The vectors were individually transformed into the *A*. *tumefaciens* strain EHA105. Transient transactivation in the leaves of *N*. *benthamiana* was performed according to the reported method [88], with minor modification. Briefly, the detached leaves of 4–week–old *N*. *benthamiana* plants were immersed into the mixture of *Agrobacterium* culture containing the effector or reporter, respectively. Then the leaves together with *Agrobacterium* culture were first subjected to sonication and later vacuum infiltrated in fresh *Agrobacterium* culture. Co–cultivation of *Agrobacterium*–infected leaves in standard 1/2 MS medium containing acetosyringone for 2 d. GUS histochemical staining was detected as described previously [89]. Primers used for DNA vectors construction are listed in S1 Table.

The promoter sequence of *OsP5CR* or *OsYUC4* was cloned into pGreenII 0800–LUC, respectively, acting as reporters. Serial constructs harboring *LUC* under the control of different regions in *OsP5CR* promoter (F1: -269~-710; F2: -787~-1117; F3–269~-1117) in the pGreenII 0800–LUC vector were generated as reporters. For *OsYUC4*, the fragment form -1486~-2139bp including CArG-box motif in *OsYUC4* promoter was used for construction of reporter. The *Renilla luciferase* (*REN*) gene driven by *35S* promoter was used as an internal control. The *OsMADS25* in pGreenII 62–SK construct described above was used as the effector. Rice shoot protoplasts were prepared and transformed using a polyethylene glycolcalcium–mediated method [90]. Firefly LUC and REN activities were surveyed with a Dual–Luciferase reporter assay kit (Promega), and the LUC activity, normalized to REN activity, was determined. All primers used for these constructs are listed in S1 Table.

### Electrophoretic mobility shift assays (EMSA)

To produce the recombinant protein, the ORF of *OsMADS25* was fused in–frame with His in pET32a and expressed in *E*. *coli* DE3 (BL21) cells, and the target recombinant OsMADS25 was purified. Oligonucleotide probes containing CArG–box motifs were synthesized and labeled with using a Biotin 3’ End DNA Labeling Kit (Thermo Scientific). For EMSA experiment assays, 30 ng of purified His-OsMADS25 recombinant protein, 400 fmol of biotin-labeled annealed oligonucleotides, 2 μl of 10× binding buffer (100 mM Tris, 500 mM KCl, and 10 mM DTT, pH 7.5), 1μl of 50% (v/v) glycerol, 1μl of 100 mM MgCl_2_, 1μl of 1 μg/μl poly (dI-dC), 1 μl of 1% (v/v) Nonidet P–40, and double–distilled water to a final volume of 20 μl. For competition assays, 20 pmol (50×) or 40 pmol (100×) of unlabeled probe was added to the reactions. The mixture was incubated at 25°C for 20 min, electrophoresed on 6% (w/v) polyacrylamide gels, and then transferred to N+ nylon membranes (Millipore). Biotin-labeled DNA was detected using the LightShift Chemiluminescent EMSA kit (Thermo Scientific, 20148).

## Accession numbers

Sequence data from this article can be found in RICEGE or GenBank/EMBL databases under the following accession number: OsMADS25 (Os04g0304400); OsRbohA (Os01g53294); OsRbohB (Os01g25820); OsRbohC (Os05g45210); OsRbohD (Os05g38980); OsRbohE (Os01g61880); OsRbohF (Os08g35210); OsRbohG (Os09g26660); OsRbohH (Os12g35610); OsRbohI (Os11g33120); OsCu/Zn–SOD (Os03g0351500); OsMn–SOD (Os05g0323900); OsCATB (Os06g0727200); OsAPX1 (Os03g0285700); OsFe–SOD (Os06g0143000); OsGR (LOC4331112); OsGTS4 (Os01g0353400); OsP5CS1 (Os05g0455500); OsP5CR (Os01g0948400); OsPOX1 (Os01g0263300); OsLEA3 (Os05g0542500); OsABI5 (Os01g0859300); OsTRAB1 (LOC4345807); OsYUC4 (Os01g0224700).

## Supporting information

S1 Fig Overexpression of *OsMADS25* enhances later root density as well as reduces ROS levels in rice leaves and bracts. **A.** Expression profile of *OsMADS25* during the growth stage (n = 3). **B.** Lateral root density of 5–day–old wild type and *OsMADS25* transgenic seedlings. **C and D.** Lateral root primordium formation in 5–day–old primary roots of wild type and *OsMADS25* transgenic lines. Scale bar, 1 mm. **E.** Shoot length of 5–day–old wild type and *OsMADS25* transgenic seedlings. **F*–*H.** Quantification of H_2_O_2_ content in the shoots of 5*–*day*–*old seedlings grown in standard 1/2 MS medium, or leaves and bracts of 2-month-old plants grown in soil. LB, leaf blade; Ro, root; LS, leaf sheath; In, inflorescence. WT, wild type. RNAi1 and RNAi2, *OsMADS25*–RNAi transgenic lines. OE1 and OE2, *OsMADS25* overexpression transgenic lines. Data are means ± SE (n = 15). The statistical significance of the measurements using one-way analysis of variance (ANOVA) was determined using Student’s *t*-test. Asterisks indicate the significant difference between *OsMADS25* transgenic lines and WT plants (*t*–test, ^*^P < 0.05, ^**^P < 0.01 or ^***^P < 0.001).
(TIF)

S2 Fig ROS accumulation in lateral root primordia of seedlings grown on standard 1/2 MS medium. Five*–*day*–*old primary roots stained by DAB or NBT to indicate O_2_^–^ and H_2_O_2_ accumulation in lateral root primordium, respectively. Scale bars, 1 mm. Red arrows indicate lateral root primordia. WT, wild type. RNAi1 and RNAi2, *OsMADS25*–RNAi transgenic lines. OE1 and OE2, *OsMADS25* overexpression transgenic lines.
(TIF)

S3 Fig *OsMADS25* does not affect the root growth in modified 1/2 MS medium. **A.** Five–day–old seedling in modified 1/2 MS medium (without nitrate, with 5 mM glutamine as the N nutrition). Scale bars, 2 cm. **B*–*D**. Measurement of primary root length, lateral root density and shoot length in image A. **E***–***F.** Lateral root primordium formation in 5–day–old primary roots. Scale bars, 1 mm. **G*–*I.** Propidium iodide (PI)–stained root epidermal cells from 5–day–old seedlings and measurement of cell length and width. Red arrows indicate lateral root primordia. WT, wild type. RNAi1 and RNAi2, *OsMADS25*–RNAi transgenic lines. OE1 and OE2, *OsMADS25* overexpression transgenic lines. Data are means ± SE (n = 15).
(TIF)

S4 Fig ROS accumulation the shoots, roots and lateral root primordia in modified 1/2 MS medium. **A*–*B**. Quantification of H_2_O_2_ content in 5*–*day*–*old shoots and roots in modified 1/2 MS medium (without nitrate, with 5 mM glutamine as the N nutrition), respectively. **C*–*D**. Primary roots stained by DAB or NBT to indicate O_2_^–^ and H_2_O_2_ accumulation in lateral root primordia in modified 1/2 MS medium, respectively. Scale bars, 1 mm. Red arrows indicate lateral root primordia. WT, wild type. RNAi1 and RNAi2, *OsMADS25*–RNAi transgenic lines. OE1 and OE2, *OsMADS25* overexpression transgenic lines. Data are means ± SE (n = 15).
(TIF)

S5 Fig Transcript levels of *OsRbohs* in roots in response to 10 mM H_2_O_2_ or 150 mM NaCl by qPCR analysis. Seedlings were grown in modified 1/2 MS medium (without nitrate, with 5 mM glutamine as the N nutrition) for 24 hours. The data represent the means ± SE of three biological replicates. Three replica experiments were performed.
(TIF)

S6 Fig Transcript levels of ROS-scavengers in roots in response to 10 mM H_2_O_2_ or 150 mM NaCl by qPCR analysis. Seedlings were grown in modified 1/2 MS medium (without nitrate, with 5 mM glutamine as the N nutrition) for 24 hours. The data represent the means ± SE of three biological replicates. Three replica experiments were performed.
(TIF)

S7 Fig *OsMADS25* reduces the sensitivity to H_2_O_2_ during seed germination as well as post-germination growth in standard 1/2 MS medium. **A** and **B**. Seven*–*day*–*old seedlings grown in standard 1/2 MS medium without or with 10 mM H_2_O_2_. Scale bars, 2 cm. **C*–*E**. Measurement of primary root length, lateral root number and shoot length shown in images A and B, respectively. **F.** Quantification of H_2_O_2_ content in the roots in images A and B. **G*–*J**. Activities of antioxidant enzymes of CAT, APX, GPX and GR in roots shown in images A and B. **K** and **L**. Comparison of seed germination in the presence of 10 mM H_2_O_2_. Scale bars, 1 cm. WT, wild type. RNAi1 and RNAi2, *OsMADS25*–RNAi transgenic lines. OE1 and OE2, *OsMADS25* overexpression transgenic lines. Data are means ± SE (n = 15*–*30). The statistical significance of the measurements using one-way analysis of variance (ANOVA) was determined using Student’s *t*-test. Asterisks indicate the significant difference between *OsMADS25* transgenic lines and WT plants (*t*–test, ^*^P < 0.05, ^**^P < 0.01 or ^***^P < 0.001).
(TIF)

S8 Fig The ROS*–*scavenging capability of *OsMADS25 in vivo*. Transient expression of *35S*::*OsMADS25* in the leaves of four–week–old *Nicotiana benthamiana* plants via *Agrobacterium*-mediated infiltration, and then treated by 150 mM NaCl for 3 days. H_2_O_2_ accumulation was indicted by DAB staining.
(TIF)

S9 Fig Expression pattern of *OsGST4* and ROS-scavenging activity of recombinant OsGST4. **A.** Sequencing result of flanking sequence and identification of insertion site in the genomic region of *osgst4*. **B.** Expression profile of *OsGST4* during the growth stage. **C.** Comparison of grains between DJ and *osgst4*, indicating blight grain rate enhanced in *osgst4*. Scale bar, 0.5 cm. D. ROS*–*scavenging capability of recombinant OsGST4 *in vitro*. (i) Growth response to H_2_O_2_ of *Escherichia coli*. (ii) Assay of ROS*–*scavenging capability of recombinant OsGST4 protein. WST, a water-soluble tetrazolium salt reagent, which can be efficiently reduced by superoxide to a stable water-soluble formazan dye with high molar absorptivity. X axis refers to the concentration of recombinant OsGST4. Data are means ± SE (n = 3). Asterisks indicate the significant difference between pET-32a-OsGST4 and pET-32a (*t*–test, ^*^P < 0.05, ^**^P < 0.01 or ^***^P < 0.001).
(TIF)

S10 Fig *OsMADS25* overexpression enhances the salt tolerance. **A.** Seed germination of wild type and *OsMADS25* transgenic lines in the presence of 150 mM NaCl. Scale bars, 1 cm. **B.** Stomatal aperture of leaves of 5-day-old seedlings observed with a scanning electron microscope. Bars, 20 μm. **C.** Frequency of open stomata. WT, wild type. RNAi1, *OsMADS25*–RNAi transgenic line. OE1, *OsMADS25* overexpression transgenic line. Data are means ± SE (n = 80–100). The statistical significance of the measurements using one-way analysis of variance (ANOVA) was determined using Student’s *t*-test. Asterisks indicate the significant difference between *OsMADS25* transgenic lines and WT plants (*t*–test, ^*^P < 0.05, ^**^P < 0.01 or ^***^P < 0.001).
(TIF)

S11 Fig Germinative growth of transgenic seedlings in the modified 1/2 MS medium supplemented with 150 mM NaCl. **A.** Three*–*day*–*old seedlings grown in modified 1/2 MS medium (without nitrate, with Gln as N nutrition) without or with 150 mM NaCl. Scale bars, 2 cm. **B*–*E.** Measurement of primary root length, lateral root number, shoot length and dry weight per plant shown in image A, respectively. **F*–*I.** Activities of antioxidant enzymes of CAT, APX, GPX and GR in shoots shown in image A. **J** and **K.** Quantification of H_2_O_2_ content of seedlings in image A. WT, wild type. RNAi1 and RNAi2, *OsMADS25*–RNAi transgenic lines. OE1 and OE2, *OsMADS25* overexpression transgenic lines. Data are means ± SE (n = 15). The statistical significance of the measurements using one-way analysis of variance (ANOVA) was determined using Student’s *t*-test. Asterisks indicate the significant difference between *OsMADS25* transgenic lines and WT plants (*t*–test, ^*^P < 0.05, ^**^P < 0.01 or ^***^P < 0.001).
(TIF)

S12 Fig Germinative growth of transgenic seedlings in standard 1/2 MS medium supplemented with 150 mM NaCl. **A.** Three*–*day*–*old seedlings grown in standard 1/2 MS medium without or with 150 mM NaCl. Scale bars, 2 cm. **B** and **C**. Measurement of the length of primary root and shoot shown in image A, respectively. **D*–*G**. Activities of antioxidant enzymes of CAT, APX, GPX and GR in shoots shown in image A. The statistical significance of the measurements using one-way analysis of variance (ANOVA) was determined using Student’s *t*-test. Asterisks indicate the significant difference between *OsMADS25* transgenic lines and WT plants (*t*–test, ^*^P < 0.05, ^**^P < 0.01 or ^***^P < 0.001). **H** and **I.** Transcript levels of ROS-producers and ROS-scavengers in seedlings exposed to NaCl in image A. The data represent the means ± SE of three biological replicates, and three replica experiments were performed. WT, wild type. RNAi1 and RNAi2, *OsMADS25*–RNAi transgenic lines. OE1 and OE2, *OsMADS25* overexpression transgenic lines. Data are means ± SE (n = 15).
(TIF)

S13 Fig Comparison of the sensitivity to ABA between wild type and transgenic lines during germinative*–*growth in standard 1/2 MS medium. **A.** Three*–*day*–*old seedlings grown in standard 1/2 MS medium without or with 5 μM ABA. Scale bars, 2 cm. **B** and **C.** Measurement of the length of primary root and shoo shown in image A, respectively. **D** and **E.** Root system stained with DAB or NBT to indicate the ROS levels under normal conditions or exposed to 5 μM ABA for 14 days. **F** and **G.** The leaves stained with DAB or NBT indicate the ROS levels exposed to 5 μM ABA for 14 days. WT, wild type. RNAi1 and RNAi2, *OsMADS25*–RNAi transgenic lines. OE1 and OE2, *OsMADS25* overexpression transgenic lines. Data are means ± SE (n = 15). The statistical significance of the measurements using one-way analysis of variance (ANOVA) was determined using Student’s *t*-test. Asterisks indicate the significant difference between *OsMADS25* transgenic lines and WT plants (*t*–test, ^*^P < 0.05, ^**^P < 0.01 or ^***^P < 0.001).
(TIF)

S14 Fig *OsYUC4* transcription is activated by OsMADS25 in *vivo*. **A.** Schematic diagrams of *OsYUC4* promoter region showing the CArG–box motif and the effector and reporter used for transient transactivation assay in rice protoplasts. *REN*, *Renilla luciferase*; *LUC*, firefly luciferase. **B.** Transactivation activity reflected by LUC activity of LUC/REN ratio. Data are means ± SE (n = 6). **C.** The transcript levels of the genes responsible for auxin biosynthesis and signaling. Data are means ± SE (n = 3). WT, wild type. RNAi1 and RNAi2, *OsMADS25*–RNAi transgenic lines. OE1 and OE2, *OsMADS25* overexpression transgenic lines. The statistical significance of the measurements using one-way analysis of variance (ANOVA) was determined using Student’s *t*-test. Asterisks indicate the significant difference between *OsMADS25* transgenic lines and WT plants (*t*–test, ^*^P < 0.05, ^**^P < 0.01 or ^***^P < 0.001).
(TIF)

S15 Fig Time course of *OsMADS25* transcription induced by 20 μM ABA via qPCR analysis. The data represent the means ± SE (n = 3). Three replica experiments were performed.
(TIF)

S1 Table Primer and probe sequences used in this study. (DOC)

S2 Table Potential targets regulated OsMADS25 might be involved in oxidative stress and growth and development. (DOCX)
