# Supplementary figures and images for: Rice transcription factor OsMADS25 modulates root growth and confers salinity tolerance via the ABA–mediated regulatory pathway and ROS scavenging
Source: PLoS Genet. 2018 Oct 10;14(10):e1007662. doi: 10.1371/journal.pgen.1007662 (PMC6197697; doi:10.1371/journal.pgen.1007662)

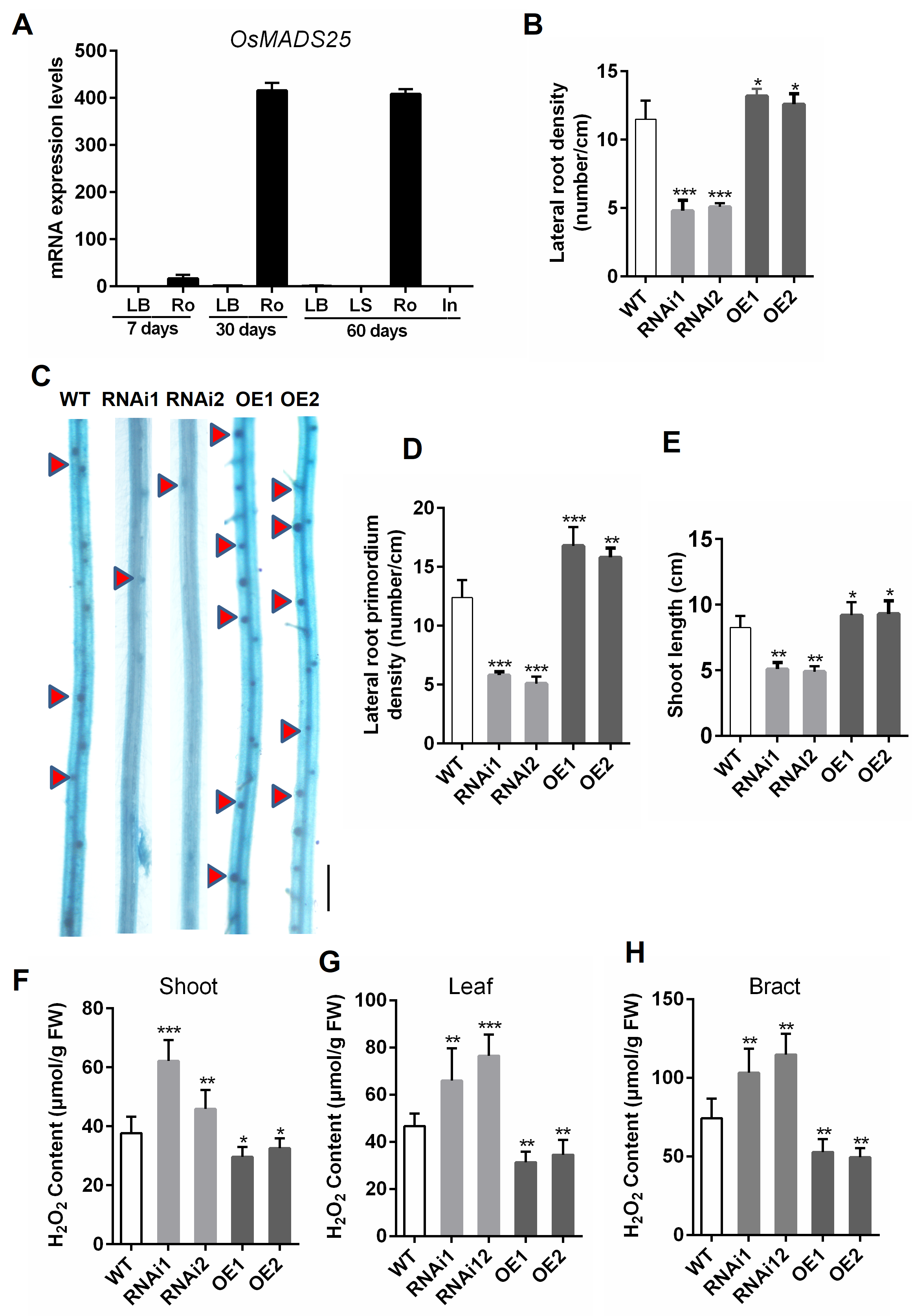

Supplement: S1 Fig — A. Expression profile of OsMADS25 during the growth stage (n = 3). B. Lateral root density of 5–day–old wild type and OsMADS25 transgenic seedlings. C and D. Lateral root primordium formation in 5–day–old primary roots of wild type and OsMADS25 transgenic lines. Scale bar, 1 mm. E. Shoot length of 5–day–old wild type and OsMADS25 transgenic seedlings. F–H. Quantification of H2O2 content in the shoots of 5–day–old seedlings grown in standard 1/2 MS medium, or leaves and bracts of 2-month-old plants grown in soil. LB, leaf blade; Ro, root; LS, leaf sheath; In, inflorescence. WT, wild type. RNAi1 and RNAi2, OsMADS25–RNAi transgenic lines. OE1 and OE2, OsMADS25 overexpression transgenic lines. Data are means ± SE (n = 15). The statistical significance of the measurements using one-way analysis of variance (ANOVA) was determined using Student’s t-test. Asterisks indicate the significant difference between OsMADS25 transgenic lines and WT plants (t–test, *P < 0.05, **P < 0.01 or ***P < 0.001). (TIF) [file pgen.1007662.s001.tif]

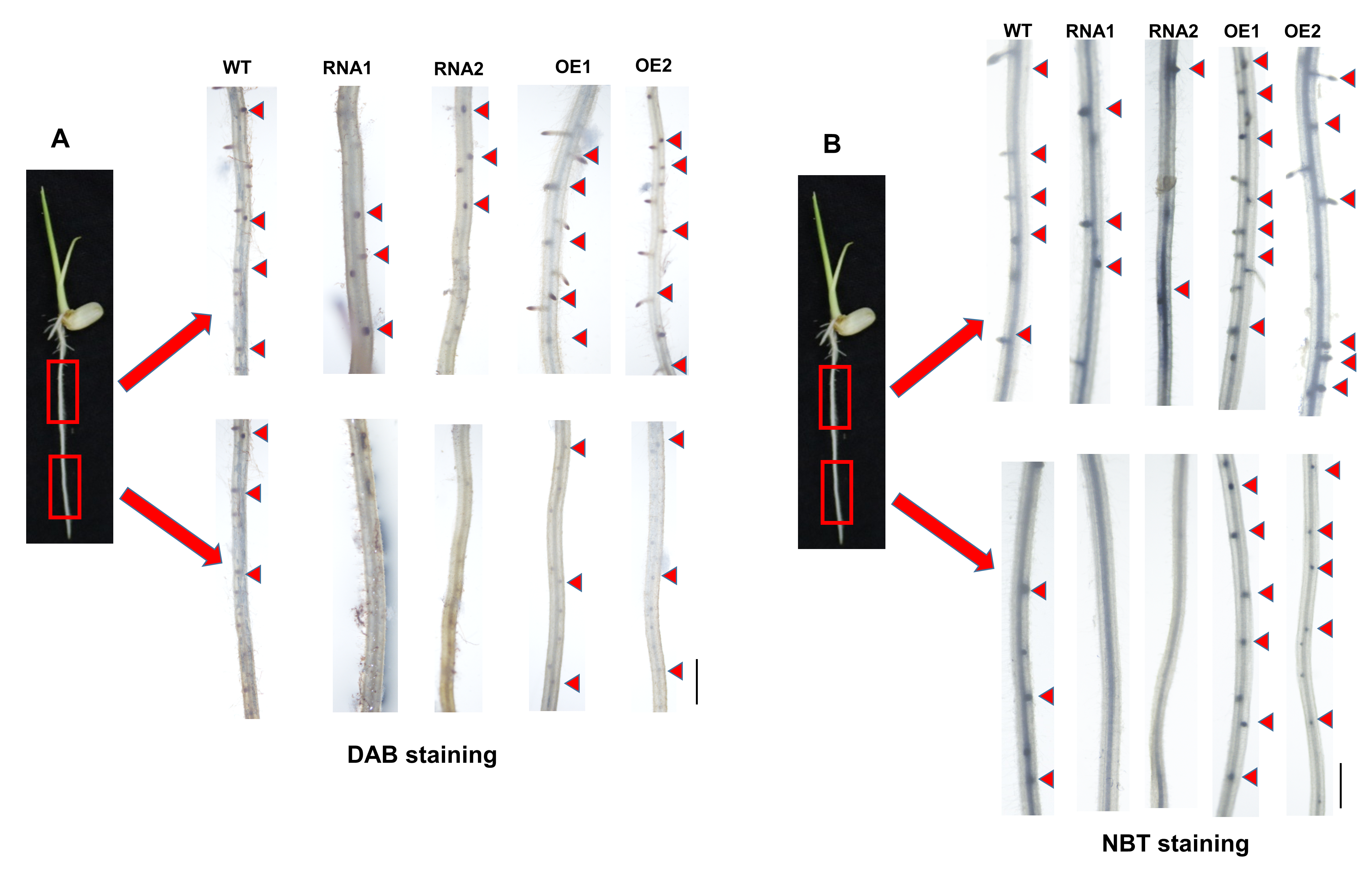

Supplement: S2 Fig — Five–day–old primary roots stained by DAB or NBT to indicate O2– and H2O2 accumulation in lateral root primordium, respectively. Scale bars, 1 mm. Red arrows indicate lateral root primordia. WT, wild type. RNAi1 and RNAi2, OsMADS25–RNAi transgenic lines. OE1 and OE2, OsMADS25 overexpression transgenic lines. (TIF) [file pgen.1007662.s002.tif]

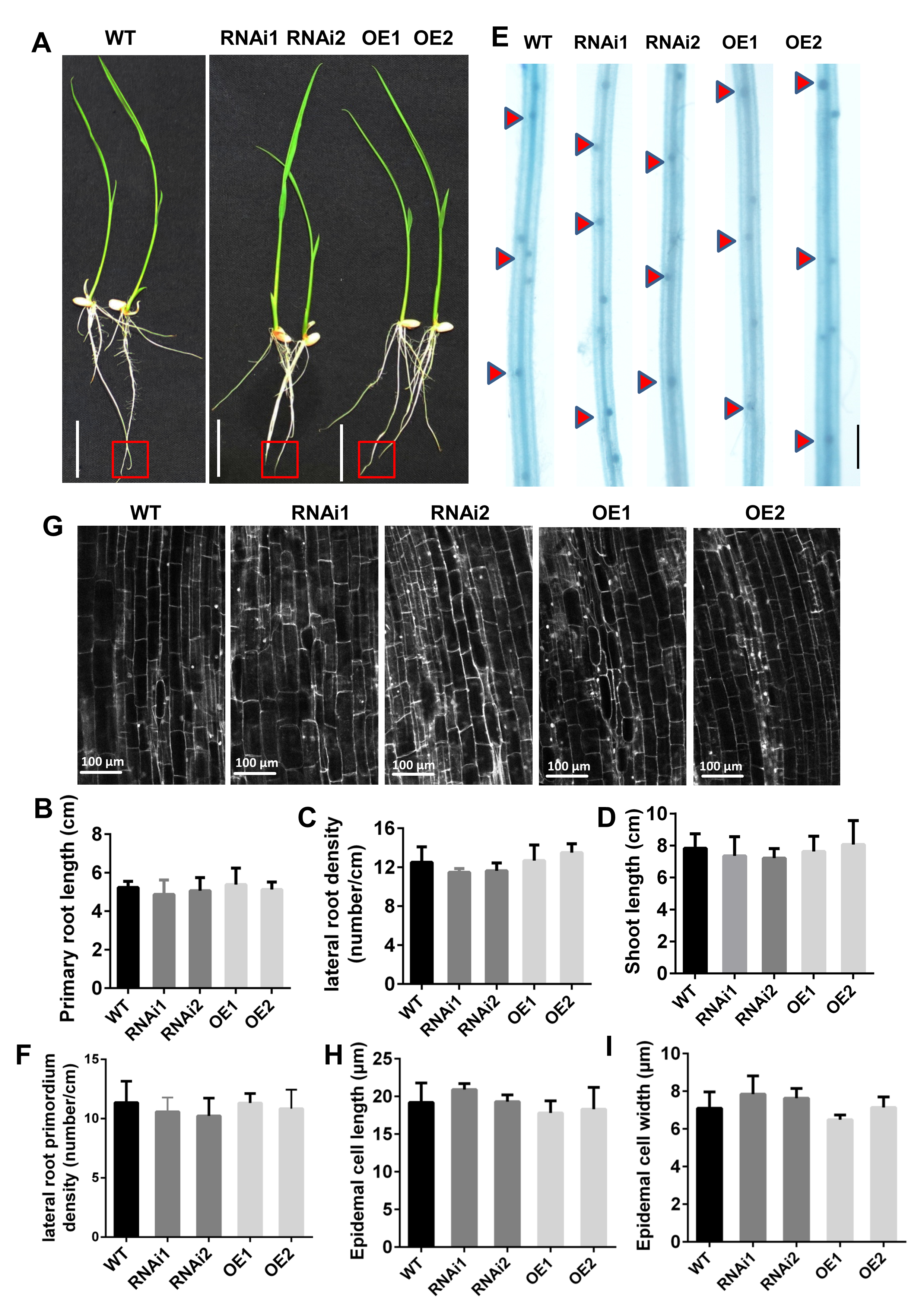

Supplement: S3 Fig — A. Five–day–old seedling in modified 1/2 MS medium (without nitrate, with 5 mM glutamine as the N nutrition). Scale bars, 2 cm. B–D. Measurement of primary root length, lateral root density and shoot length in image A. E–F. Lateral root primordium formation in 5–day–old primary roots. Scale bars, 1 mm. G–I. Propidium iodide (PI)–stained root epidermal cells from 5–day–old seedlings and measurement of cell length and width. Red arrows indicate lateral root primordia. WT, wild type. RNAi1 and RNAi2, OsMADS25–RNAi transgenic lines. OE1 and OE2, OsMADS25 overexpression transgenic lines. Data are means ± SE (n = 15). (TIF) [file pgen.1007662.s003.TIF]

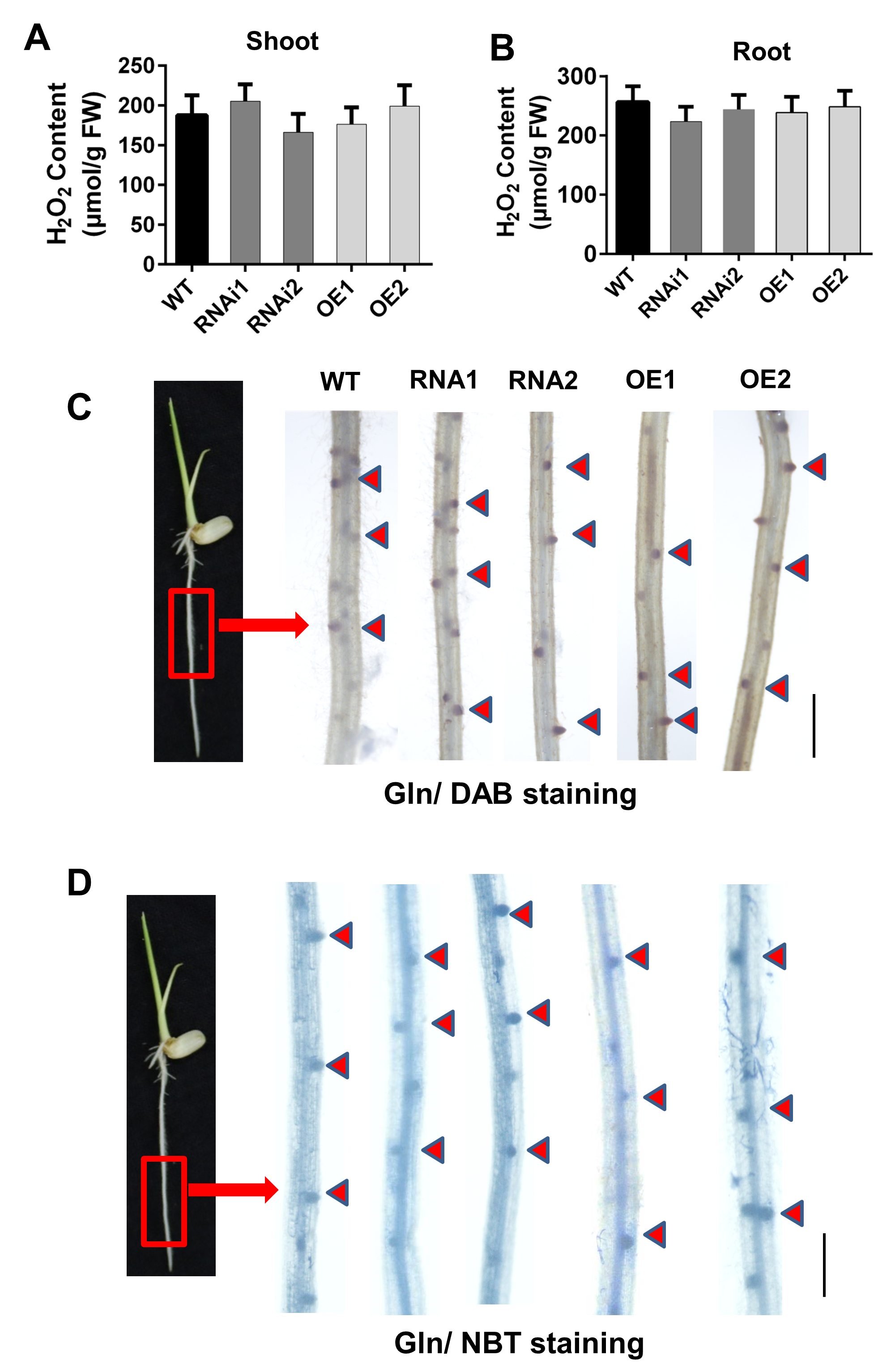

Supplement: S4 Fig — A–B. Quantification of H2O2 content in 5–day–old shoots and roots in modified 1/2 MS medium (without nitrate, with 5 mM glutamine as the N nutrition), respectively. C–D. Primary roots stained by DAB or NBT to indicate O2– and H2O2 accumulation in lateral root primordia in modified 1/2 MS medium, respectively. Scale bars, 1 mm. Red arrows indicate lateral root primordia. WT, wild type. RNAi1 and RNAi2, OsMADS25–RNAi transgenic lines. OE1 and OE2, OsMADS25 overexpression transgenic lines. Data are means ± SE (n = 15). (TIF) [file pgen.1007662.s004.tif]

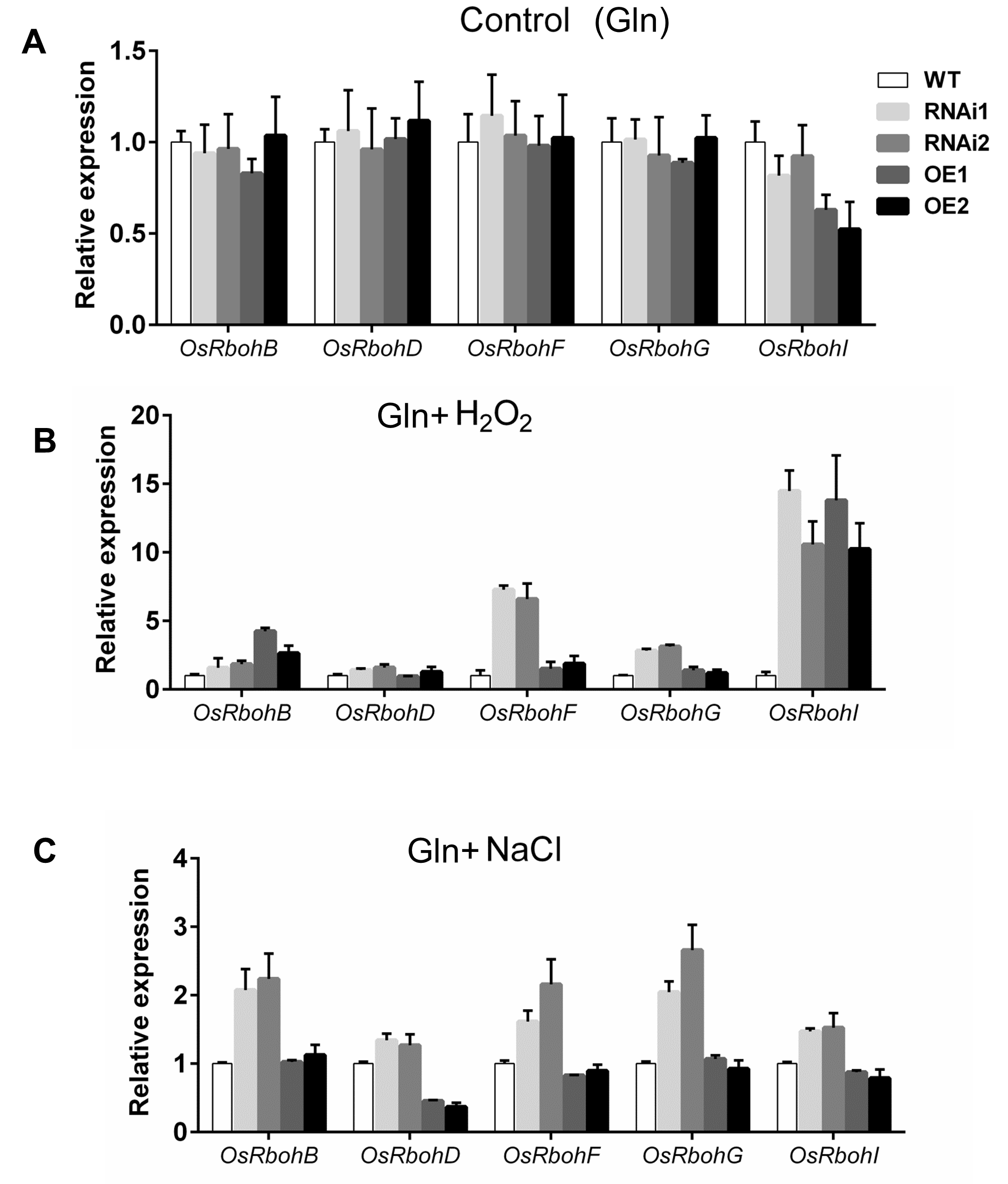

Supplement: S5 Fig — Seedlings were grown in modified 1/2 MS medium (without nitrate, with 5 mM glutamine as the N nutrition) for 24 hours. The data represent the means ± SE of three biological replicates. Three replica experiments were performed. (TIF) [file pgen.1007662.s005.tif]

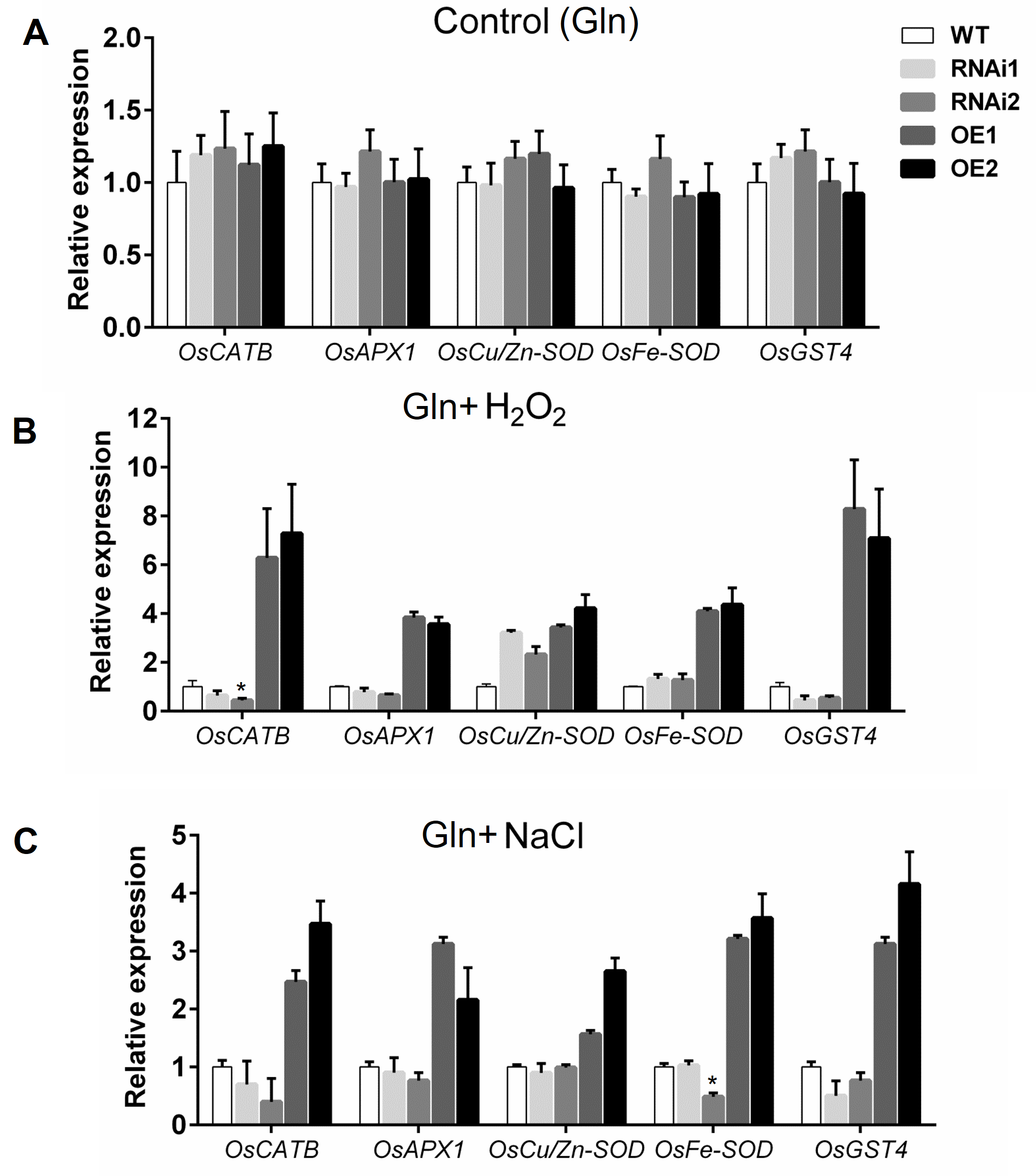

Supplement: S6 Fig — Seedlings were grown in modified 1/2 MS medium (without nitrate, with 5 mM glutamine as the N nutrition) for 24 hours. The data represent the means ± SE of three biological replicates. Three replica experiments were performed. (TIF) [file pgen.1007662.s006.tif]

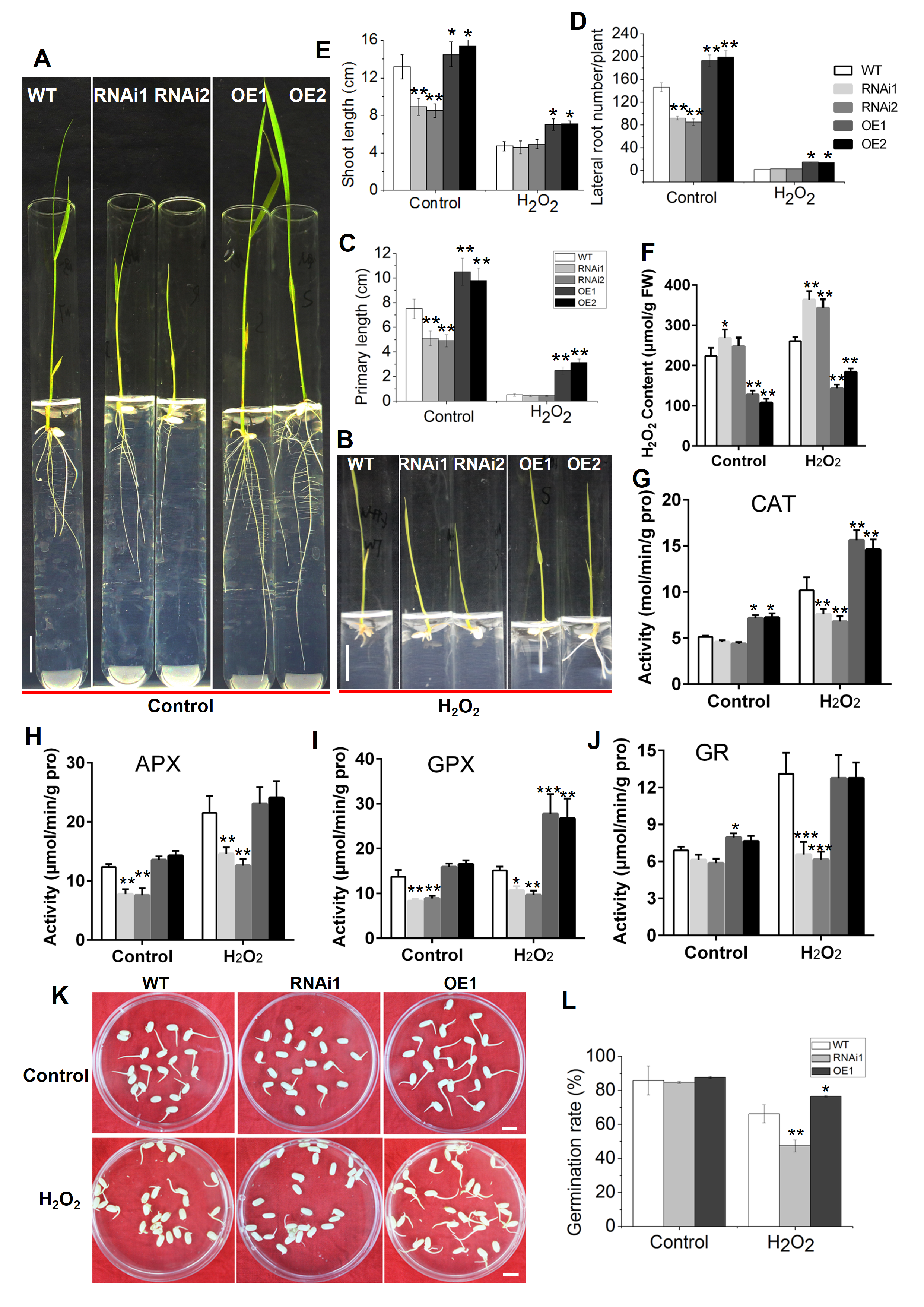

Supplement: S7 Fig — A and B. Seven–day–old seedlings grown in standard 1/2 MS medium without or with 10 mM H2O2. Scale bars, 2 cm. C–E. Measurement of primary root length, lateral root number and shoot length shown in images A and B, respectively. F. Quantification of H2O2 content in the roots in images A and B. G–J. Activities of antioxidant enzymes of CAT, APX, GPX and GR in roots shown in images A and B. K and L. Comparison of seed germination in the presence of 10 mM H2O2. Scale bars, 1 cm. WT, wild type. RNAi1 and RNAi2, OsMADS25–RNAi transgenic lines. OE1 and OE2, OsMADS25 overexpression transgenic lines. Data are means ± SE (n = 15–30). The statistical significance of the measurements using one-way analysis of variance (ANOVA) was determined using Student’s t-test. Asterisks indicate the significant difference between OsMADS25 transgenic lines and WT plants (t–test, *P < 0.05, **P < 0.01 or ***P < 0.001). (TIF) [file pgen.1007662.s007.TIF]

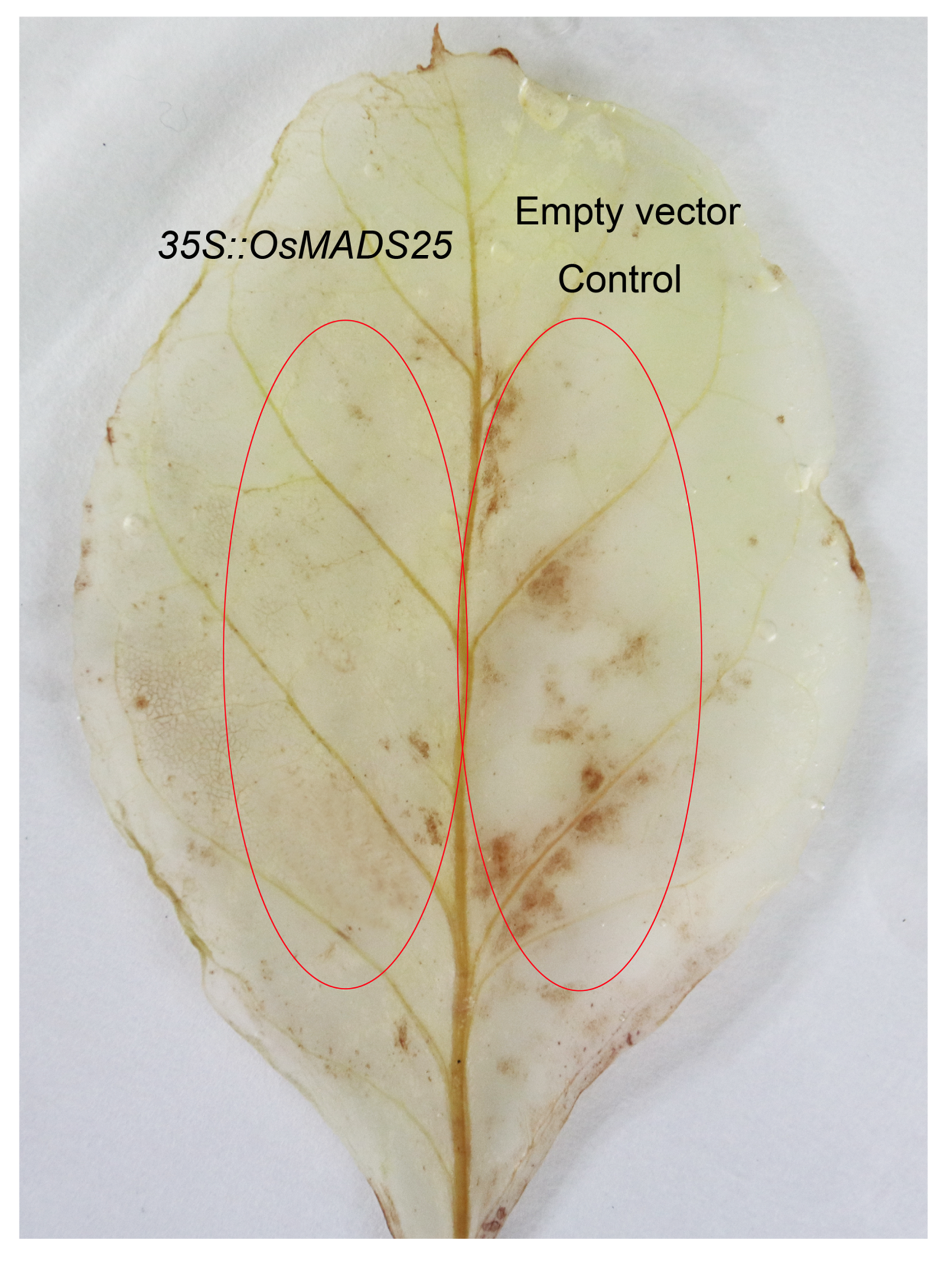

Supplement: S8 Fig — Transient expression of 35S::OsMADS25 in the leaves of four–week–old Nicotiana benthamiana plants via Agrobacterium-mediated infiltration, and then treated by 150 mM NaCl for 3 days. H2O2 accumulation was indicted by DAB staining. (TIF) [file pgen.1007662.s008.TIF]

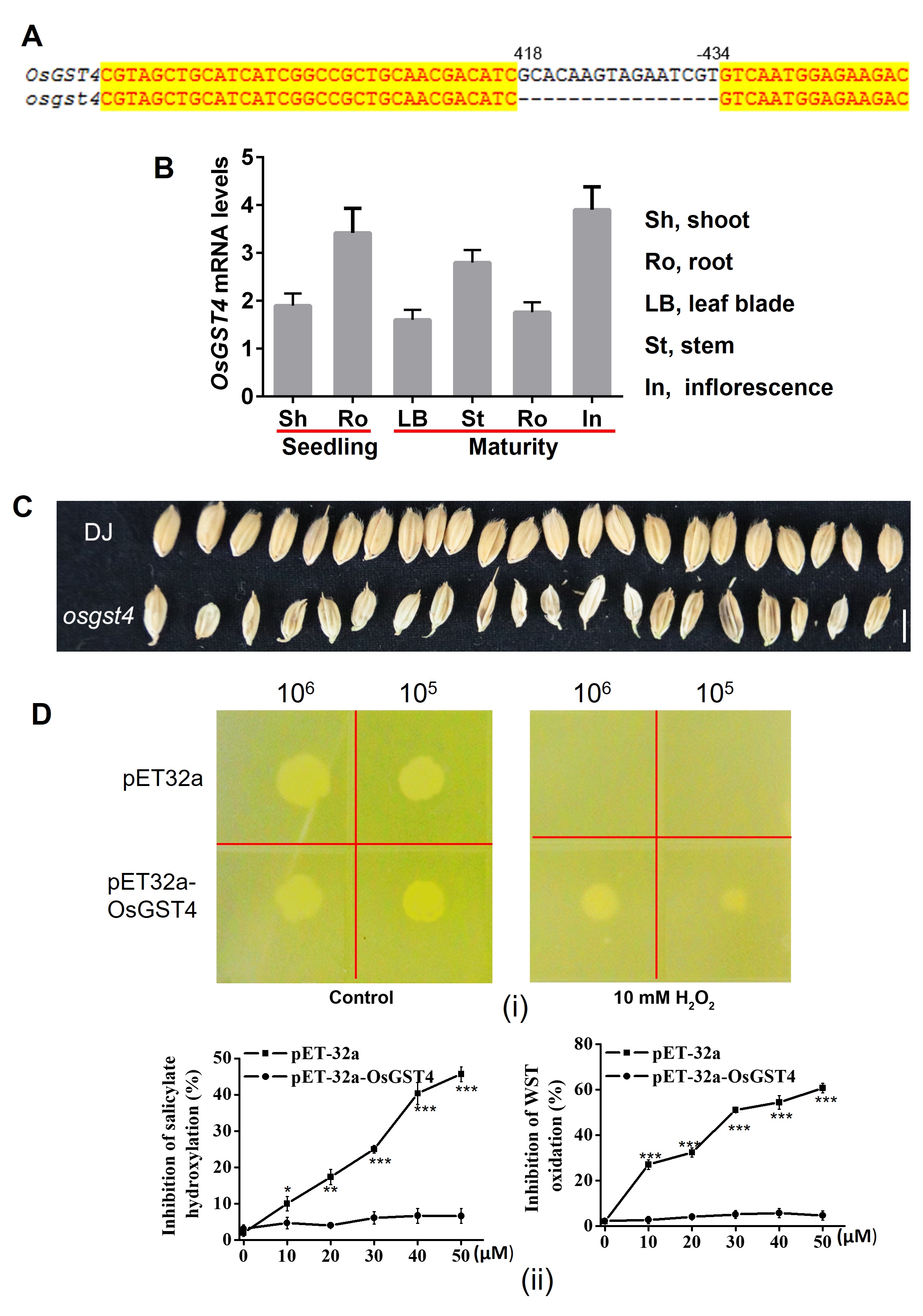

Supplement: S9 Fig — A. Sequencing result of flanking sequence and identification of insertion site in the genomic region of osgst4. B. Expression profile of OsGST4 during the growth stage. C. Comparison of grains between DJ and osgst4, indicating blight grain rate enhanced in osgst4. Scale bar, 0.5 cm. D. ROS–scavenging capability of recombinant OsGST4 in vitro. (i) Growth response to H2O2 of Escherichia coli. (ii) Assay of ROS–scavenging capability of recombinant OsGST4 protein. WST, a water-soluble tetrazolium salt reagent, which can be efficiently reduced by superoxide to a stable water-soluble formazan dye with high molar absorptivity. X axis refers to the concentration of recombinant OsGST4. Data are means ± SE (n = 3). Asterisks indicate the significant difference between pET-32a-OsGST4 and pET-32a (t–test, *P < 0.05, **P < 0.01 or ***P < 0.001). (TIF) [file pgen.1007662.s009.TIF]

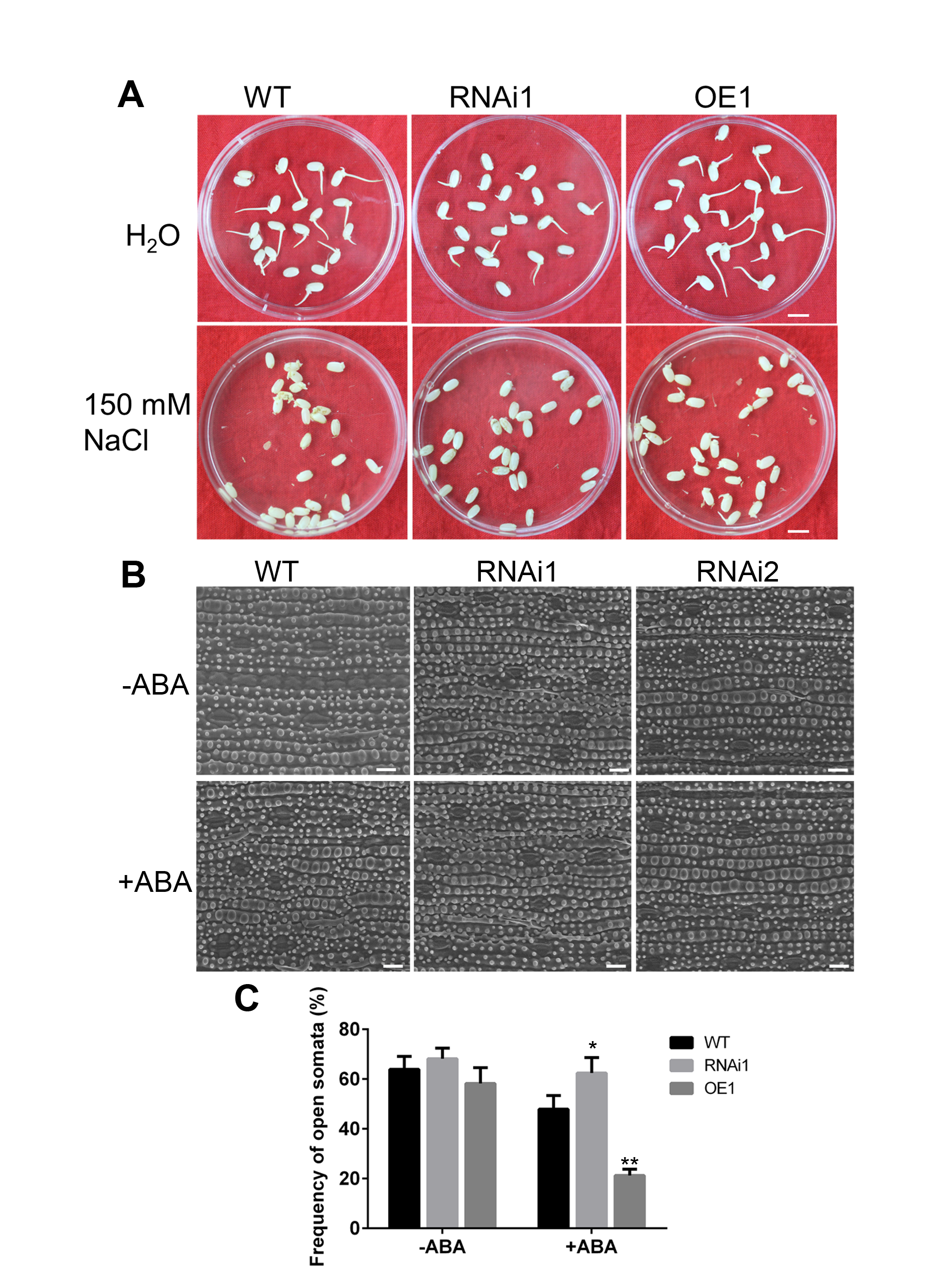

Supplement: S10 Fig — A. Seed germination of wild type and OsMADS25 transgenic lines in the presence of 150 mM NaCl. Scale bars, 1 cm. B. Stomatal aperture of leaves of 5-day-old seedlings observed with a scanning electron microscope. Bars, 20 μm. C. Frequency of open stomata. WT, wild type. RNAi1, OsMADS25–RNAi transgenic line. OE1, OsMADS25 overexpression transgenic line. Data are means ± SE (n = 80–100). The statistical significance of the measurements using one-way analysis of variance (ANOVA) was determined using Student’s t-test. Asterisks indicate the significant difference between OsMADS25 transgenic lines and WT plants (t–test, *P < 0.05, **P < 0.01 or ***P < 0.001). (TIF) [file pgen.1007662.s010.TIF]

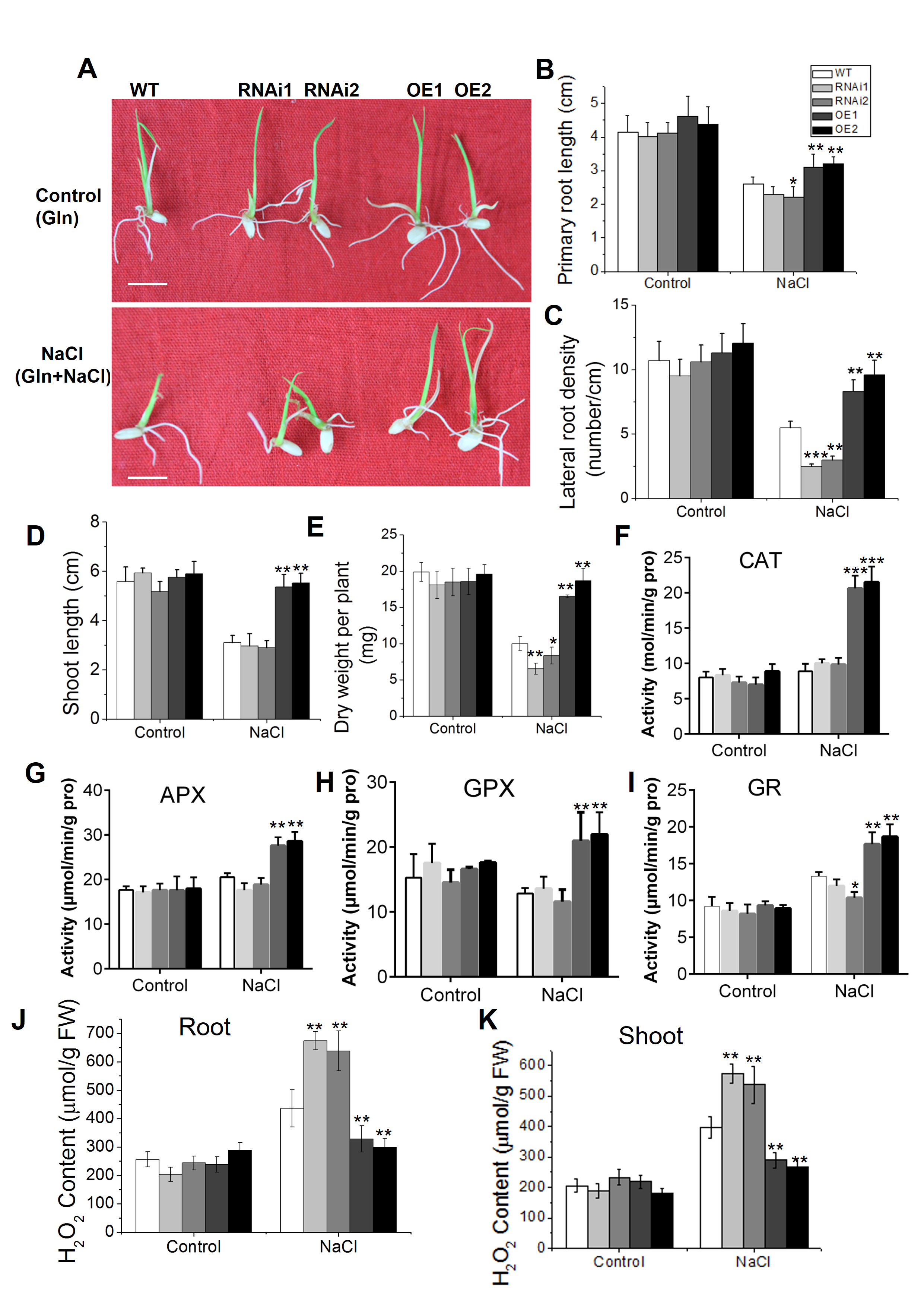

Supplement: S11 Fig — A. Three–day–old seedlings grown in modified 1/2 MS medium (without nitrate, with Gln as N nutrition) without or with 150 mM NaCl. Scale bars, 2 cm. B–E. Measurement of primary root length, lateral root number, shoot length and dry weight per plant shown in image A, respectively. F–I. Activities of antioxidant enzymes of CAT, APX, GPX and GR in shoots shown in image A. J and K. Quantification of H2O2 content of seedlings in image A. WT, wild type. RNAi1 and RNAi2, OsMADS25–RNAi transgenic lines. OE1 and OE2, OsMADS25 overexpression transgenic lines. Data are means ± SE (n = 15). The statistical significance of the measurements using one-way analysis of variance (ANOVA) was determined using Student’s t-test. Asterisks indicate the significant difference between OsMADS25 transgenic lines and WT plants (t–test, *P < 0.05, **P < 0.01 or ***P < 0.001). (TIF) [file pgen.1007662.s011.TIF]

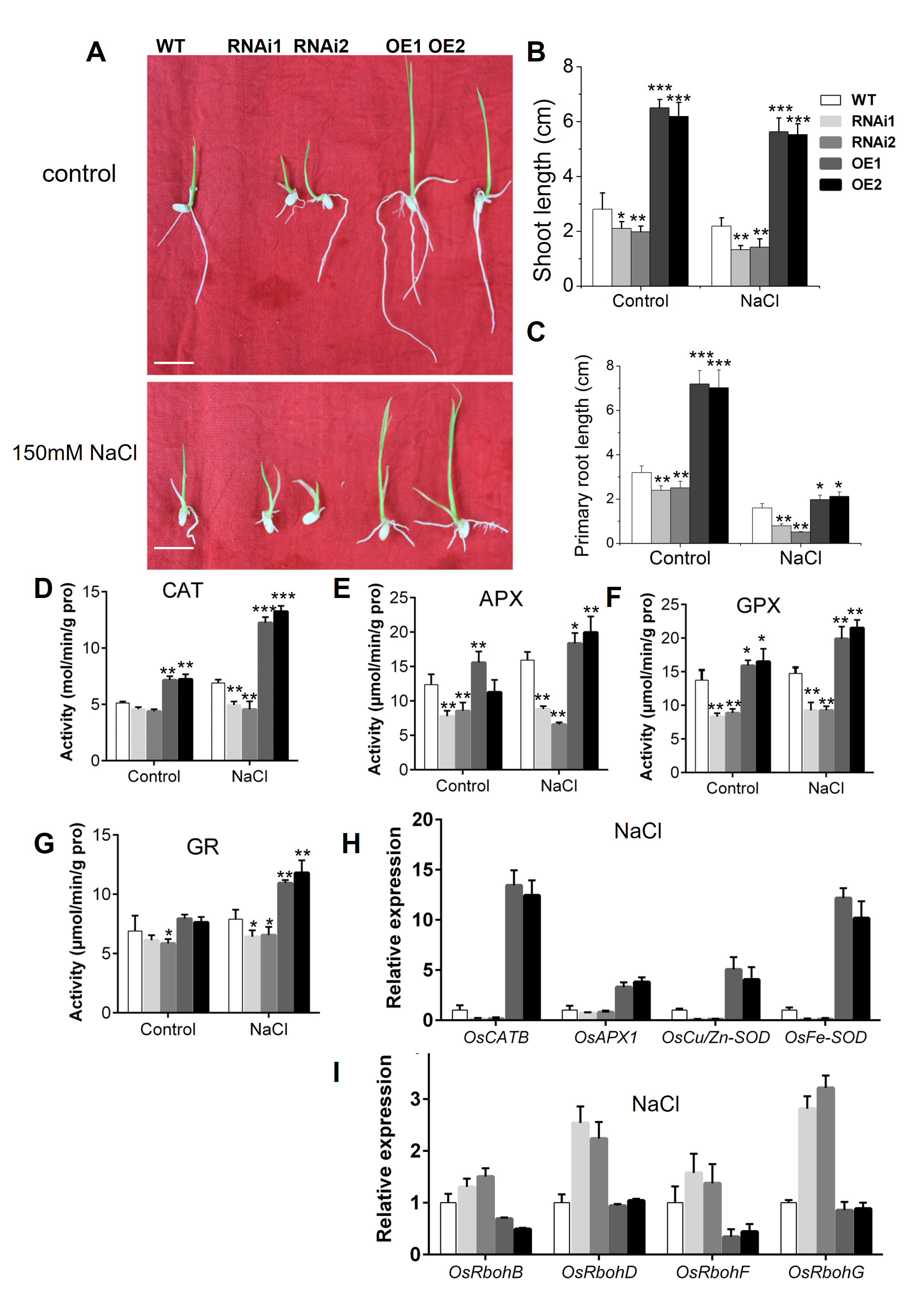

Supplement: S12 Fig — A. Three–day–old seedlings grown in standard 1/2 MS medium without or with 150 mM NaCl. Scale bars, 2 cm. B and C. Measurement of the length of primary root and shoot shown in image A, respectively. D–G. Activities of antioxidant enzymes of CAT, APX, GPX and GR in shoots shown in image A. The statistical significance of the measurements using one-way analysis of variance (ANOVA) was determined using Student’s t-test. Asterisks indicate the significant difference between OsMADS25 transgenic lines and WT plants (t–test, *P < 0.05, **P < 0.01 or ***P < 0.001). H and I. Transcript levels of ROS-producers and ROS-scavengers in seedlings exposed to NaCl in image A. The data represent the means ± SE of three biological replicates, and three replica experiments were performed. WT, wild type. RNAi1 and RNAi2, OsMADS25–RNAi transgenic lines. OE1 and OE2, OsMADS25 overexpression transgenic lines. Data are means ± SE (n = 15). (TIF) [file pgen.1007662.s012.TIF]

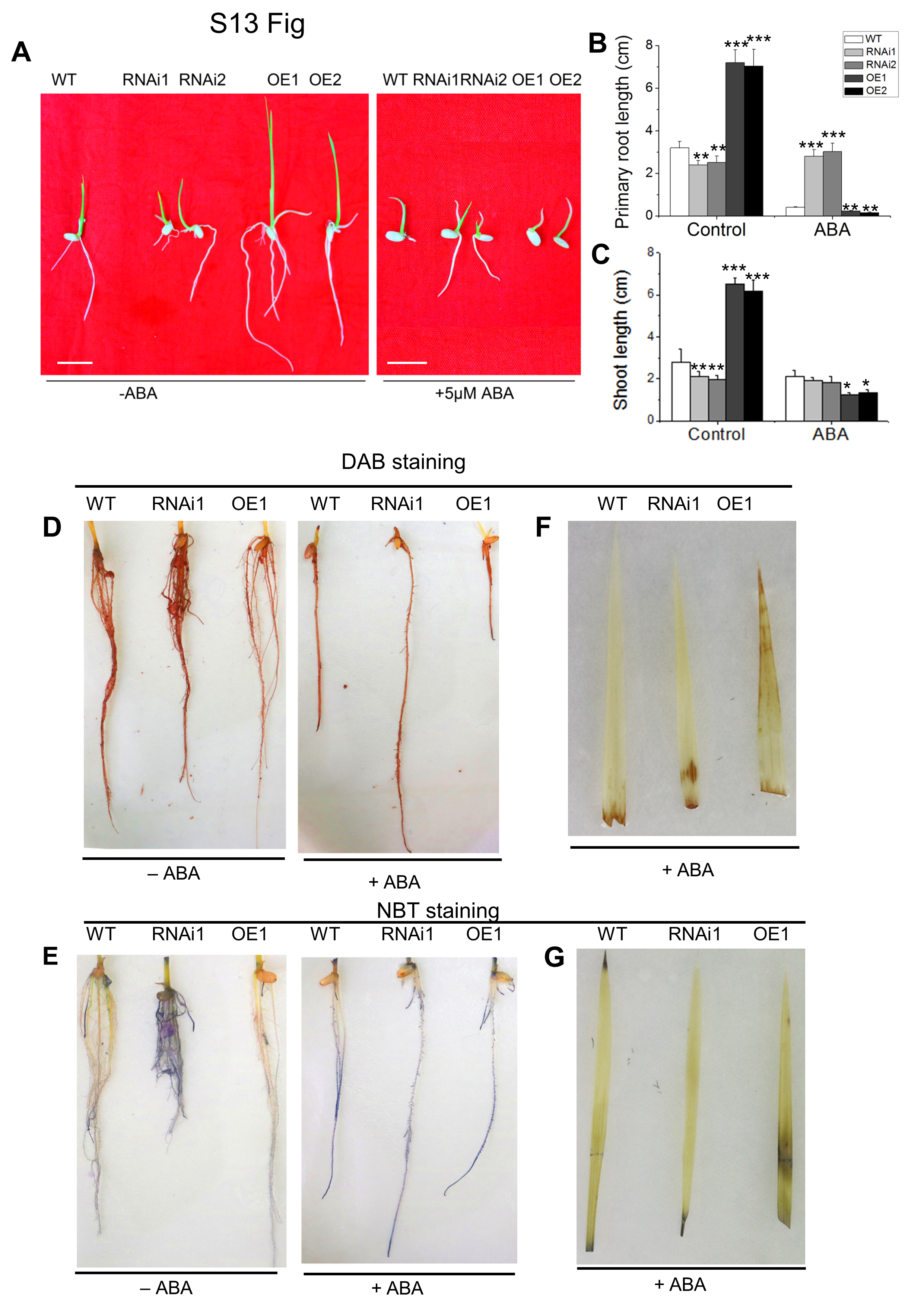

Supplement: S13 Fig — A. Three–day–old seedlings grown in standard 1/2 MS medium without or with 5 μM ABA. Scale bars, 2 cm. B and C. Measurement of the length of primary root and shoo shown in image A, respectively. D and E. Root system stained with DAB or NBT to indicate the ROS levels under normal conditions or exposed to 5 μM ABA for 14 days. F and G. The leaves stained with DAB or NBT indicate the ROS levels exposed to 5 μM ABA for 14 days. WT, wild type. RNAi1 and RNAi2, OsMADS25–RNAi transgenic lines. OE1 and OE2, OsMADS25 overexpression transgenic lines. Data are means ± SE (n = 15). The statistical significance of the measurements using one-way analysis of variance (ANOVA) was determined using Student’s t-test. Asterisks indicate the significant difference between OsMADS25 transgenic lines and WT plants (t–test, *P < 0.05, **P < 0.01 or ***P < 0.001). (TIF) [file pgen.1007662.s013.TIF]

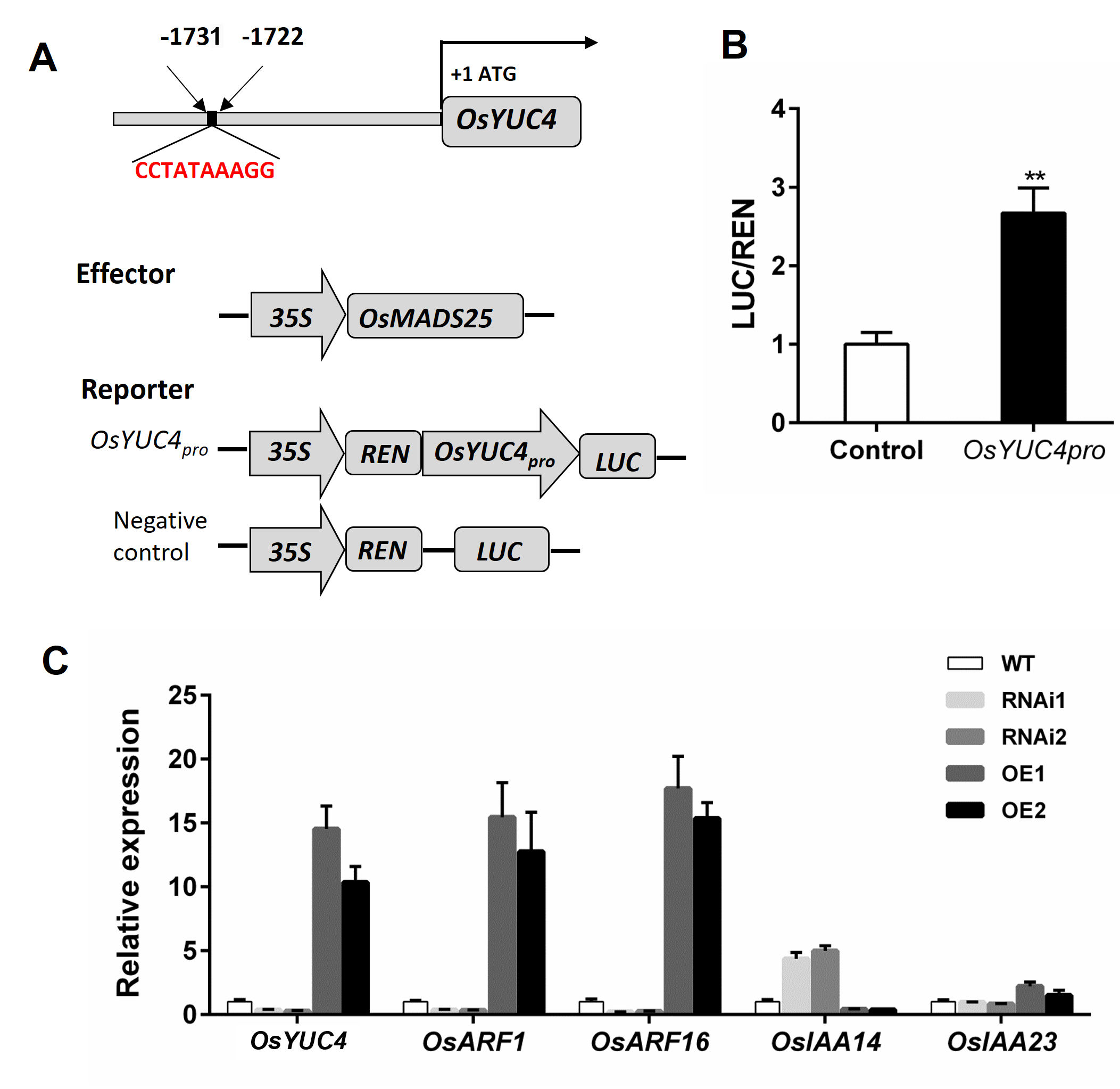

Supplement: S14 Fig — A. Schematic diagrams of OsYUC4 promoter region showing the CArG–box motif and the effector and reporter used for transient transactivation assay in rice protoplasts. REN, Renilla luciferase; LUC, firefly luciferase. B. Transactivation activity reflected by LUC activity of LUC/REN ratio. Data are means ± SE (n = 6). C. The transcript levels of the genes responsible for auxin biosynthesis and signaling. Data are means ± SE (n = 3). WT, wild type. RNAi1 and RNAi2, OsMADS25–RNAi transgenic lines. OE1 and OE2, OsMADS25 overexpression transgenic lines. The statistical significance of the measurements using one-way analysis of variance (ANOVA) was determined using Student’s t-test. Asterisks indicate the significant difference between OsMADS25 transgenic lines and WT plants (t–test, *P < 0.05, **P < 0.01 or ***P < 0.001). (TIF) [file pgen.1007662.s014.tif]

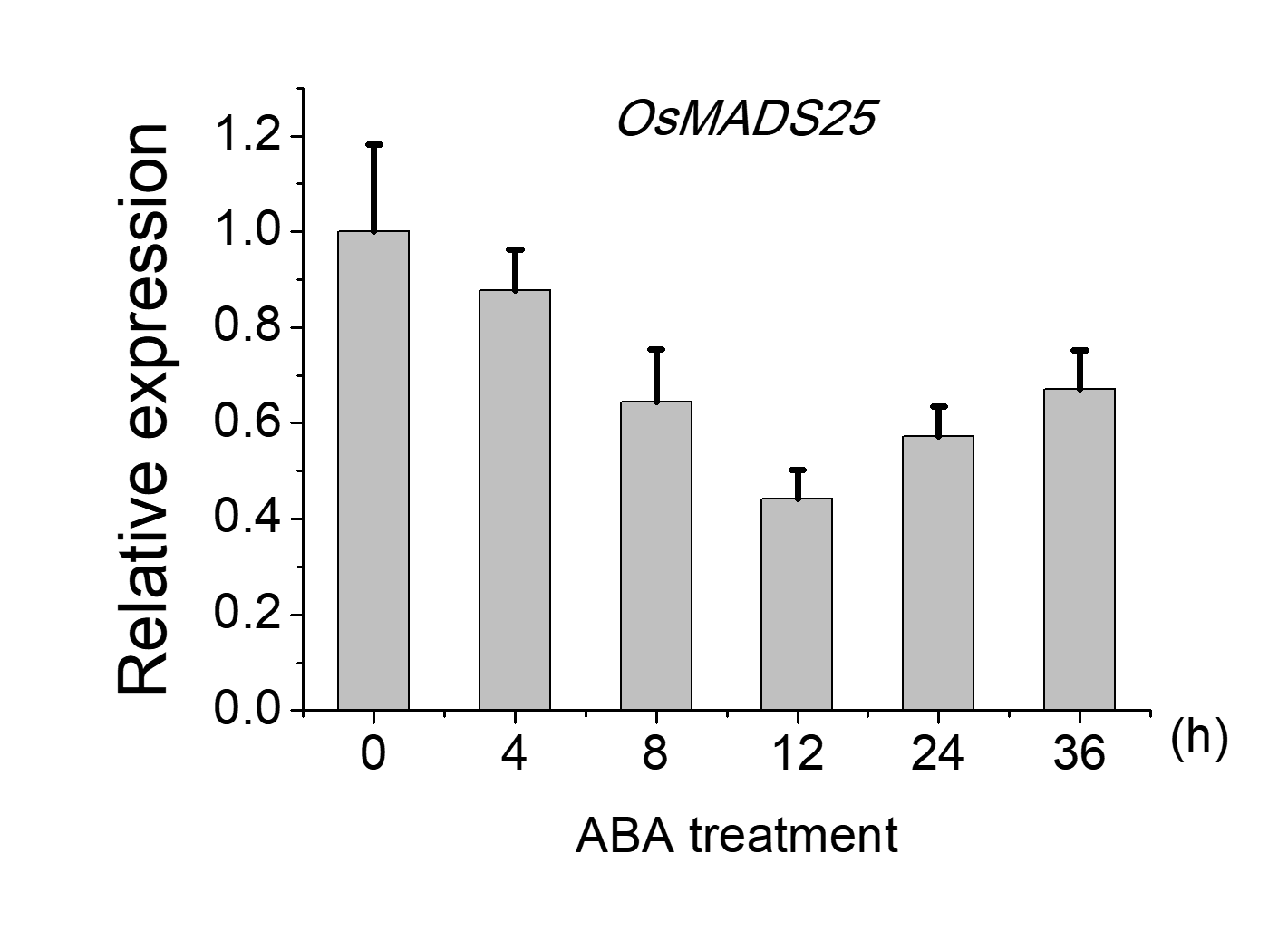

Supplement: S15 Fig — The data represent the means ± SE (n = 3). Three replica experiments were performed. (TIF) [file pgen.1007662.s015.TIF]
