# Supplementary material for: Rice transcription factor OsMADS25 modulates root growth and confers salinity tolerance via the ABA–mediated regulatory pathway and ROS scavenging
Source: PLoS Genet. 2018 Oct 10;14(10):e1007662. doi: 10.1371/journal.pgen.1007662 (PMC6197697; doi:10.1371/journal.pgen.1007662)
Supplement: S1 Table — (DOC) [file pgen.1007662.s016.doc]

Table 1 Primers and probes used in this study

| **Primers for qRT-PCR** (The underline showed the restriction enzyme sites. F, forward; R, reverse) | | | | | | |
| --- | --- | --- | --- | --- | --- | --- |
| Name | Sequences(5’ -3’) | | | | | Experiments |
| OsMADS25 –F/R | TGAACCCAATGTCAGAGGCTA / TCCTTTTGAACAAGAGACCCC | | | | | qRT-PCR |
| OsRbohA –F/R | gtcttatgcagtcatgaatgtaca / gaataatatacagttaattagcct | | | | | qRT-PCR |
| OsRbohB –F/R | cctagtggaagaagctgtgct / cactatgaaagggaacatcacaa | | | | | qRT-PCR |
| OsRbohC –F/R | tgttttagggatggttttacac / tgtacagacagaaggttaacgt | | | | | qRT-PCR |
| OsRbohD –F/R | gaccagaccaggaaaaaaacaccaa/acacagaaagagttgctaaccgatg | | | | | qRT-PCR |
| OsRbohE –F/R | catcgtgcatagattctgga / catgcattcccactgttcca | | | | | qRT-PCR |
| OsRbohF –F/R | tcgtctatcatagatatacatg / cgtgtactttggtgacctcag | | | | | qRT-PCR |
| OsRbohG –F/R | aagcgttgctaattttcgctat / gagaggatgtttttttgaacgg | | | | | qRT-PCR |
| OsRbohH –F/R | gtacaattatatacagattaatg / cgaacaaccaatcactcactaa | | | | | qRT-PCR |
| OsRbohI–F/R | tggccagataatttcatcggtt / gctactctaagtattacaaagta | | | | | qRT-PCR |
| OsCu/Zn-SOD-F/R | CTTGCGGGATCATCGGACTT / GAGCTCGTAGAGTTTCAGGCT | | | | | qRT-PCR |
| OsMn-SOD -F/R | GCCAGACTACCTGAGCAACA/ CGTTGCCTTCAGACAACATCA | | | | | qRT-PCR |
| OsCATB -F/R | GTTCGGTTCTCCACAGTCGT/ CCCTCCATGTGCCTGTAGTT | | | | | qRT-PCR |
| OsGR -F/R | AACAGCCGATGGCATAAAAG/ CAACCACCAGTTTCATGACG | | | | | qRT-PCR |
| OsAPX1-F/R | CCAAGGGTTCTGACCACCTA/ CAGTTCGGAGAGCTTGAGGT | | | | | qRT-PCR |
| OsGST4-1-F/R | TCACAGCGACGGAGTACGGC/ GGCATGCCGGCGATCACCTT | | | | | qRT-PCR |
| OsP5CS1-F/R | GTGGAGGAGGAGAGGCTG/ TGCCCAAAGCCAATCTTC | | | | | qRT-PCR |
| OsP5CR-F/R | AATAGAGGCCATGGCTGATG/ AATGCACCCTTCTCAAGCTC-3 | | | | | qRT-PCR |
| OsLEA3- F/R | GCAGTACACCAAGGACTCTGC/ ACCCAAAGGGAAATCATTCAC | | | | | qRT-PCR |
| OsABI5- F/R | GAAGAATTCCAGGCTACCACC/ TTGATCTCAGTCCACACCTCC | | | | | qRT-PCR |
| β-actin-F/R | AGGAAGGCTGGAAGAGGACC/ CGGGAAATTGTGAGGGACAT | | | | | qRT-PCR |
| Ubiquitin -F/R | CGAGTCTAACGGACACCA/ AAAGATGACCCGACAAAC | | | | | qRT-PCR |
| OsTRAB1- F/R | ATATGGATTCAGCGGGCAAGGG/ TCCACCACCTTCTCCACGTTAC | | | | | qRT-PCR |
| OsZIP23- F/R | GGAGCTGAACGATGAACTCCAG/ TCGGCTCATTCTCTCTAGAACCTC | | | | | qRT-PCR |
| OsZIP46- F/R | GAACACTGACTGGTCCATGCTG/ GAGAGAAGCAACTCTGAAGCTGAG | | | | | qRT-PCR |
| OsMADS25-F/R | AGCACGTCAAGTGAGCAAGT/ AGTGGTGCAATACGCTACACA | | | | | RT-PCR |
| RT-EF1α-F/ R | GTACAAGATCGGTGGTATT/ GGGTACTCAGAGAAGGTCT | | | | | RT-PCR |
| OsGTS4-P1/P2 | TGGGAACTAAAAAGATGCCG/ TTCAGGTGCTGATGATGACC | | | | | OsGST4 mutant screening |
| P3/P4 | CCACAGTTTTCGCGATCCAGACTG/ TTGGGGTTTCTACAGGACGTAAC | | | | |
| **Primers for generating DNA vector** | | | | | | |
| Name | | Sequences(5’ -3’) | | Experiments | | |
| OsMADS25- F | | ATGAGCTCGCCAGGGTTTTCCCAGTC | | PCAMBIA1301, overexpression | | |
| OsMADS25- R | | GAGTCGACGAATTGTGTTTCCATGATCTAAGTC | | PCAMBIA1301, overexpression | | |
| OsMAD25 -D-topo-F | | CACCTGCTCATCAACGCAACATAGAA | | D-topo, RNAi | | |
| OsMAD25 -D-topo-R | | AAATTCAGTGGTGCAATACGCT | | D-topo, RNAi | | |
| OsMADS25- His-F | | CCATGGCTATGGGGAGAGGGAAG | | pET32a, Recombiant protein, EMSA | | |
| OsMADS25- His-R | | CATATGTTATTCATCTTCAACTTC | | pET32a, Recombiant protein, EMSA | | |
| OsGTS4-His-F | | GAATTCATGCTGGTGTGCCTGGAG | | pET32a, Recombiant protein, ROS scavenging activity | | |
| OsGST4-His-R | | CTCGAGCTATGGCAAGTTCCCACTTC | | pET32a, Recombiant protein, ROS scavenging activity | | |
| 35S-GFP-F | | GAGCGGCCGCATGGTGAGCAAGGGCGAGGAG | | pGreenII 62-SK, transient expression | | |
| 35S-GFP-R | | GCTCTAGATCAAAGATCTACCATGTACAGCTCGT | | pGreenII 62-SK, transient expression | | |
| 35S-OsMADS25-F | | ATGAGCTCGCCAGGGTTTTCCCAGTC | | pGreenII 62-SK,transient expression | | |
| 35S-OsMADS25-R | | GAGTCGACGAATTGTGTTTCCATGATCTAAGTC | | pGreenII 62-SK,transient expression | | |
| 35S- OsGTS4pro-F | | GGGGTACCCcagattgagatagcagatcg | | pGreenII 0800-GUS, transient expression | | |
| 35S- OsGTS4pro-R | | GCTCTAGAGCTAGAAGATCCCAACCCTCC | | pGreenII 0800-GUS, transient expression | | |
| 35S- OsP5CRpro-P1-F | | CTCGAGAGTACGGTAGTAGCATCCAA | | pGreenII 0800, transient expression | | |
| 35S- OsP5CRpro-P1-R | | GATATCGCCAGGAGCTCGTATGAGAG | | pGreenII 0800, transient expression | | |
| 35S- OsP5CRpro-P2-F | | CTCGAGTTCCACTATGAGTAGTAAGTGGC | | pGreenII 0800, transient expression | | |
| 35S- OsP5CRpro-P2-R | | GATATCTGCGATAAGAACAGGCTCCC | | pGreenII 0800, transient expression | | |
| 35S- OsP5CRpro-P3-F | | CTCGAGAGTACGGTAGTAGCATCCAA | | pGreenII 0800, transient expression | | |
| 35S- OsP5CRpro-P3-R | | GATATCTGCGATAAGAACAGGCTCCC | | pGreenII 0800, transient expression | | |
| YOsGTS4-up | | GGGGTACCCCCTCCTAAATCAAGTCATGCTTGCATGCTGTGATCAATTAATAGTTGACCAGCCGCTAGTTGCCGGCGCCCTCGAGGG | | Yeast one-hybrid | | |
| YOsGTS4-down | | CCCTCGAGGGCGCCGGCAACTAGCGGCTGGTCAACTATTAATTGATCACAGCATGCAAGCATGACTTGATTTAGGAGGGGGTACCCC | | Yeast one-hybrid | | |
| Mut-YOsGTS4-up | | GGGGTACCCCCTCCTAAATCAAGTCATGCTTGCATGCTGTGATAAATTAATATTTGACCAGCCGCTAGTTGCCGGCGCCCTCGAGGG | | Yeast one-hybrid | | |
| Mut-YOsGTS4-down | | CCCTCGAGGGCGCCGGCAACTAGCGGCTGGTCAAATATTAATTTATCACAGCATGCAAGCATGACTTGATTTAGGAGGGGGTACCCC | | Yeast one-hybrid | | |
| **Probes for EMSA** | | | | | | |
| Name | | | Sequences(5’ -3’) | | Experiments | |
| OsGTS4-sense | | | CATGCTGTGATCAATTAATAGTTGACCAGCC | | EMSA | |
| OsGTS4-anti sense | | | GGCTGGTCAACTATTAATTGATCACAGCATG | | EMSA | |
| Mut-OsGTS4-sense | | | CATGCTGTGATAAATTAATATTTGACCAGCC | | EMSA | |
| Mut-OsGTS4-anti sense | | | GGCTGGTCAAATATTAATTTATCACAGCATG | | EMSA | |
| OsP5CR-P1-sense | | | cagagggcattctccctaataaggcagatacctccg | | EMSA | |
| OsP5CR-P1-antisense | | | CGGAGGTATCTGCCTTATTAGGGAGAATGCCCTCTG | | EMSA | |
| Mut-OsP5CR-P1-sense | | | cagagggcattctcTTtaataaAAcagatacctccg | | EMSA | |
| Mut-OsP5CR-P1-antisense | | | CGGAGGTATCTGTTTTATTAAAGAGAATGCCCTCTG | | EMSA | |
| OsP5CR-P2-sense | | | cgacgaatccattcatatatttgtaggggcagcgg | | EMSA | |
| OsP5CR-P2-antisense | | | CCGCTGCCCCTACAAATATATGAATGGATTCGTCG | | EMSA | |
| Mut-OsP5CR-P2-sense | | | cgacgaatccattTatatatttAtaggggcagcgg | | EMSA | |
| Mut-OsP5CR-P2-antisense | | | CCGCTGCCCCTATAAATATATAAATGGATTCGTCG | | EMSA | |
