# Supplementary material for: Rice transcription factor OsMADS25 modulates root growth and confers salinity tolerance via the ABA–mediated regulatory pathway and ROS scavenging
Source: PLoS Genet. 2018 Oct 10;14(10):e1007662. doi: 10.1371/journal.pgen.1007662 (PMC6197697; doi:10.1371/journal.pgen.1007662)
Supplement: S2 Table — (DOCX) [file pgen.1007662.s017.docx]

**S2 Table Potential targets of OsMADS25 involved in oxidative stress and growth and development**

| **RAP_Locus** | **MSU_Locus** | **Fold Enrichment** | **Annotation** | **Function or putative functions** |
| --- | --- | --- | --- | --- |
| **Biotic and abiotic stress** | | | | |
| Os01g0353400 | LOC_Os01g25100 | 8.96 | Glutathione s-transferase putative, expressed (OsGST4) | ROS |
| Os01g0368900 | [LOC_Os01g27140](http://rice.plantbiology.msu.edu/cgi-bin/ORF_infopage.cgi?orf=LOC_Os01g27140) | 34.72 | Glutaredoxin（OsGRX4） | ROS |
| Os02g0240100 | [LOC_Os02g14430](http://rice.plantbiology.msu.edu/cgi-bin/ORF_infopage.cgi?orf=LOC_Os02g14430) | 18.7 | Peroxidase precursor, putative, expressed | ROS |
| Os11g0284900 | [LOC_Os11g18170](http://rice.plantbiology.msu.edu/cgi-bin/ORF_infopage.cgi?orf=LOC_Os11g18170) | 13.15 | Glutathione peroxidase, putative, expressed | ROS |
| Os04g0134800 | [LOC_Os04g04750](http://rice.plantbiology.msu.edu/cgi-bin/ORF_infopage.cgi?orf=LOC_Os04g04750) | 17.57 | Peroxidase precursor, putative, expressed | ROS |
| Os04g0171600 | [LOC_Os04g08824](http://rice.plantbiology.msu.edu/cgi-bin/ORF_infopage.cgi?orf=LOC_Os04g08824) | 13.29 | Cytochrome P450, putative, expressed | ROS |
| Os01g0502400 | [LOC_Os01g31770](http://rice.plantbiology.msu.edu/cgi-bin/ORF_infopage.cgi?orf=LOC_Os01g31770) | 25.62 | Oxidoreductase, 2OG-Fe oxygenase family protein, putative, expressed | ROS |
| Os06g0234200 | [LOC_Os06g12790](http://rice.plantbiology.msu.edu/cgi-bin/ORF_infopage.cgi?orf=LOC_Os06g12790) | 7.59 | Rac-like GTP-binding protein 4 | ROS |
| Os04g0388500 | [LOC_Os04g31900](http://rice.plantbiology.msu.edu/cgi-bin/ORF_infopage.cgi?orf=LOC_Os04g31900) | 4.49 | Calmodulin-binding transcription activator 4, putative, expressed | Ca2+ signaling |
| Os03g0711300 | [LOC_Os03g50330](http://rice.plantbiology.msu.edu/cgi-bin/ORF_infopage.cgi?orf=LOC_Os03g50330) | 16.56 | CAMK_KIN1/SNF1/Nim1_like.18, CAMK includes calcium/calmodulin depedent protein kinases, expressed | Ca2+ signaling |
| Os03g0808600 | [LOC_Os03g59390](http://rice.plantbiology.msu.edu/cgi-bin/ORF_infopage.cgi?orf=LOC_Os03g59390) | 28.64 | CAMK_CAMK_like.24, CAMK includes calcium/calmodulin depedent protein kinases, expressed | Ca2+ signaling |
| Os01g0809300 | [LOC_Os01g59440](http://rice.plantbiology.msu.edu/cgi-bin/ORF_infopage.cgi?orf=LOC_Os01g59440) | 6.27 | Extracellular leucine-rich repeat domain protein(OsLRR1) | Disease responsive |
| Os01g0831200 | [LOC_Os01g61500](http://rice.plantbiology.msu.edu/cgi-bin/ORF_infopage.cgi?orf=LOC_Os01g61500) | 13.98 | Bcl-2-associated athanogene (BAG) protein(OsBAG4) | Disease Resistance |
| Os03g0667100 | [LOC_Os03g46440](http://rice.plantbiology.msu.edu/cgi-bin/ORF_infopage.cgi?orf=LOC_Os03g46440) | 13.23 | Disease resistance(OsNPR3) | Disease Resistance |
| Os12g0511500 | [LOC_Os12g32670](http://rice.plantbiology.msu.edu/cgi-bin/ORF_infopage.cgi?orf=LOC_Os12g32670) | 9.49 | CC-NBS-LRR resistance protein, putative, expressed | Disease Resistance |
| Os11g0271100 | [LOC_Os11g17080](http://rice.plantbiology.msu.edu/cgi-bin/ORF_infopage.cgi?orf=LOC_Os11g17080) | 10.44 | Mitogen-activated protein kinase OsMPK15 | Chilling stress |
| Os02g0527300 | [LOC_Os02g32590](http://rice.plantbiology.msu.edu/cgi-bin/ORF_infopage.cgi?orf=LOC_Os02g32590) | 33.91 | Heat shock transcription factor gene（OsHsfA3） | Temperature and oxidative stresses |
| Os02g0624300 | [LOC_Os02g41510](http://rice.plantbiology.msu.edu/cgi-bin/ORF_infopage.cgi?orf=LOC_Os02g41510) | 21.26 | cold-responsive R2R3-type MYB gene（OsMYB30） | Chilling stress |
| Os01g0948400 | [LOC_Os01g71990](http://rice.plantbiology.msu.edu/cgi-bin/ORF_infopage.cgi?orf=LOC_Os01g71990) | 24.47 | [pyrroline-5-carboxylate reductase](http://rice.plantbiology.msu.edu/cgi-bin/ORF_infopage.cgi?orf=LOC_Os01g71990)（*OsP5CR*） | Salt stress and ABA responsive |
| **Phytohormone** | | | | |
| Os02g0698800 | [LOC_Os02g47060](http://rice.plantbiology.msu.edu/cgi-bin/ORF_infopage.cgi?orf=LOC_Os02g47060) | 1.88 | WRKY transcription factor（OsWRKY66） | Abscisic Acid Signaling |
| Os03g0797800 | [LOC_Os03g58350](http://rice.plantbiology.msu.edu/cgi-bin/ORF_infopage.cgi?orf=LOC_Os03g58350) | 29.55 | *OsIAA14* | Auxin-responsive |
| Os01g0224700 | [LOC_Os01g12490](http://rice.plantbiology.msu.edu/cgi-bin/ORF_infopage.cgi?orf=LOC_Os01g12490) | 19.57 | *OsYUCCA4* | Auxin Biosynthesis |
| [Os12g0512000](http://rapdb.dna.affrc.go.jp/viewer/gbrowse_details/irgsp1?name=Os12g0512000) | [LOC_Os12g32750](http://rice.plantbiology.msu.edu/cgi-bin/ORF_infopage.cgi?orf=LOC_Os12g32750) | 9.49 | *OsYUCCA5* | Auxin Biosynthesis |
| Os02g0771600 | [LOC_Os02g53180](http://rice.plantbiology.msu.edu/cgi-bin/ORF_infopage.cgi?orf=LOC_Os02g53180) | 11.58 | aminocyclopropane-1-carboxylate oxidase gene（OsACO3） | Ethylene Biosynthesis and auxin related |
| [**Growth and development**](javascript:;) | | | | |
| Os01g0831000 | [LOC_Os01g61480](http://rice.plantbiology.msu.edu/cgi-bin/ORF_infopage.cgi?orf=LOC_Os01g61480) | 13.98 | LAX1 | Spikelet Development |
| Os01g0900400 | [LOC_Os01g67430](http://rice.plantbiology.msu.edu/cgi-bin/ORF_infopage.cgi?orf=LOC_Os01g67430) | 23.55 | lipase gene(EG1) | Spikelet Development |
| Os10g0189100 | [LOC_Os10g11140](http://rice.plantbiology.msu.edu/cgi-bin/ORF_infopage.cgi?orf=LOC_Os10g11140) | 12.73 | plastidic phosphoglucomutase(OspPGM) | Starch synthesis and male sterility |
| Os03g0650400 | [LOC_Os03g44760](http://rice.plantbiology.msu.edu/cgi-bin/ORF_infopage.cgi?orf=LOC_Os03g44760) | 9.69 | homolog of Arabidopsis SWI1 and maize AM1（OsAM1） | Meiosis |

Note: target genes with the cis–element of CArG–box regulated by OsMADS25 by ChIP–seq analysis
